# Participatory Scenario Design to Support Ex-ante Biodiversity and Ecosystem Services Assessments in Four European Agricultural Case Studies

**DOI:** 10.1007/s00267-026-02435-y

**Published:** 2026-04-01

**Authors:** Katrin Karner, Monika Suškevičs, Florian Danzinger, Sonja Kay, Claudia Bethwell, Noëlle Klein, Takamasa Nishizawa, Johannes Schuler, Michael Glemnitz, Peter Zander, Tobias Conradt, Kalev Sepp, Maaria Semm, Laura Hämäläinen, Janar Raet, Rando Värnik, Miguel Villoslada, Thomas Wrbka, Martin Schönhart

**Affiliations:** 1 BOKU University, Institute of Sustainable Economic Development, Department of Economics and Social Sciences, Vienna, Feistmantelstraße 4, 1180 Vienna, Austria; 2 Estonian University of Life Sciences, Institute of Agricultural and Environmental Sciences, Kreutzwaldi 1, 51006 Tartu, Estonia; 3 Department of Botany and Biodiversity Research, University of Vienna, Rennweg 14, A-1030 Vienna, Austria; 4 Agroscope, Integrative Agroecology, Reckenholzstrasse 191, 8046 Zürich, Switzerland; 5 Leibniz Centre for Agricultural Landscape Research (ZALF) e.V., Eberswalder Str. 84, 15374 Müncheberg, Germany; 6 Geography Department, Humboldt-Universitat zu Berlin, Unter den Linden 6, 10099 Berlin, Germany; 7 Chair of Planning of Landscape and Urban Systems (PLUS), Institute for Spatial and Landscape Planning, Department of Civil, Environmental and Geomatic Engineering, ETH Zurich, Stefano-Franscini-Platz 5, 8093 Zurich, Switzerland; 8 Potsdam Institute for Climate Impact Research, Telegrafenberg A26, 14473 Potsdam, Germany; 9 Department of Geographical and Historical Studies, Faculty of Social Sciences and Business Studies, University of Eastern Finland, Yliopistokatu 7, Metria, 3th floor, Joensuu, Finland; 10 Federal Institute of Agricultural Economics, Rural and Mountain Research, Dietrichgasse 27, 1030 Vienna, Austria

**Keywords:** Stakeholder engagement, global change, land use drivers, scenario design, agricultural land use, nature conservation

## Abstract

Anticipating future socioeconomic conditions through scenarios supports effective land-use planning and management that safeguards biodiversity and ecosystem services (BES). This study introduces a novel participatory scenario development protocol to design consistent, nested regional scenarios tailored for BES assessments of agricultural land-use. The protocol is applied in four European case studies (subnational regions in Austria, Estonia, Germany, and Switzerland), combining regional narratives with quantitative developments aligned with the Shared Socioeconomic Pathways for European agri-food systems for 2050 (Eur-Agri-SSPs). Two innovative scenario components are introduced: (i) land-use and management practices and (ii) land-use-biodiversity actions, including private and public instruments. These components are typically neglected in larger-scale scenario applications. Despite shared European boundary conditions from the Eur-Agri-SSPs, the regional scenarios exhibit substantial variation, driven by current land-use structures and stakeholder input. Scenario elements, shaped by existing funding schemes and socioeconomic contexts, vary substantially across scenarios and regions. Examples include the share of organic farms and the level of payments for agri-environment-climate measures. In Münsterland (Germany) and Lääne County (Estonia), current agri-environment payments are significantly lower than in Schwarzbubenland (Switzerland) or the Wienerwald (Austria), and this is reflected in the SSP1 (“..sustainable paths”) and SSP2 (“..established paths”) scenarios. This study demonstrates the value of regional extensions of the SSP framework, grounded in participatory processes, to support context-specific BES assessments. It contributes to scenario research by presenting key challenges and recommendations for nested participatory scenario design and by bridging the gap between global and continental frameworks and subnational implementation needs.

## Introduction

Biodiversity loss remains one of the most urgent environmental challenges globally, driven by land-use change, climate change, pollution, and the spread of invasive species (IPBES, 2019; Jaureguiberry et al., 2022; Keck et al., 2025). In response, several European strategies, such as the EU Biodiversity Strategy for 2030, aim to reverse these trends by, e.g., building a coherent trans-European nature network and halving pesticide use (European Commission, 2020). Similarly, the EU’s Zero Pollution Action Plan envisions clean air, water, and soil for all (European Commission, 2021). Despite these ambitions, the pathways to achieving such goals—and managing the trade-offs involved—remain unclear.

To effectively safeguard biodiversity and ecosystem services (BES) in agricultural landscapes, policies must anticipate future socioeconomic and climate conditions. Scenario approaches are critical tools in this regard, offering structured ways to explore plausible futures, inform planning, and manage uncertainty (Kok et al., 2015, 2006; van der Hejden, 2005; Walker et al., 2003; Wiebe et al., 2018).

The climate change research community has developed Shared Socioeconomic Pathways (SSPs) – narratives of global development that support assessments of climate change impacts, mitigation, and adaptation, and corresponding private and public decision-making. The SSPs distinguish pathways regarding the degree of “climate change adaptation” as well as “climate change mitigation” and describe respective developments in six topics, i.e., demographics, human development, economy and lifestyle, policies and institutions, technology, environment and natural resources (SSPs: O’Neill et al., 2017; Eur-SSPs: Kok et al., 2019; Eur-Agri-SSPs: Mitter et al., 2020). The global SSPs were so far extended to include details for several thematic contexts and downscaled to different spatial units, ranging from individual countries (e.g., Tschumi et al., 2026) to transnational geographical areas (e.g., Palazzo et al., 2017). For instance, Kok et al. (2019) adapted and matched the “CLIMSAVE” scenarios ex-post with the global SSPs in a participatory manner, leading to SSPs for Europe (Eur-SSPs: Kok et al., 2019; Pedde et al., 2019). Mitter et al. (2020) developed “Shared socioeconomic pathways for European agriculture and food systems (Eur-Agri-SSPs)”. The authors engaged with diverse stakeholders across Europe to identify drivers relevant to agriculture and food systems and to describe how these drivers change (increase, decrease, or remain constant) in the downscaled SSPs. They used the global SSPs as boundary conditions (Zurek and Henrichs, 2007) and applied a protocol (Mitter et al., 2019) to ensure consistency between the global and European scales.

Although the SSPs have become a cornerstone in global environmental change research (O'Neill et al., 2020), they have been criticized for **one key limitation: their limited applicability in BES assessments** (Kok et al., 2017; Pereira et al., 2020; Rosa et al., 2020) due to insufficient detail on land management practices and their limited representation of the diverse human values that underpin relationships with nature (Pereira et al., 2020; Rosa et al., 2020). Although the Eur-Agri-SSPs extended the SSPs for agriculture and food systems, including human-nature interactions, they still lack the spatial resolution and institutional specificity required to support BES assessments effectively. Thematic extensions of the Eur-Agri-SSPs so far focused on pest management (Nagesh et al., 2023). Despite these limitations, the SSP framework provides a suitable foundation for further extensions due to its structured logic, cross-scale consistency, and compatibility with integrated modeling frameworks that combine ecological and economic components (such as highlighted by Engstr**ö**m et al., 2016).

Nevertheless, studies often develop their own scenarios or scenario frameworks when assessing BES (e.g., Martins et al., 2020; Mosnier et al., 2022; Mouchet et al., 2017; Priess et al., 2018; Zerriffi et al., 2022). This makes comparisons between, e.g., impact studies difficult because they do not share the same or at least similar scenarios or are not based on the same key assumptions. Exceptions, where the well-established SSPs and RCPs (Representative concentration pathways, describing the development of greenhouse gas emissions and respective global warming; Meinshausen et al., 2011; van Vuuren et al., 2011) are combined and applied to assess BES at the global scale, are provided for instance by Powers and Jetz (2019), Rabin et al. (2020), or Lecl**è**re et al. (2020). Such global-scale studies are valuable for demonstrating magnitudes and relationships among climate change, land-use policies, and markets, and for confirming that consideration of both climate and socioeconomic change is required when assessing BES.

**A second key limitation of existing SSP applications is their marginal connection to the spatial contexts in which biodiversity-relevant decisions are made and measured**, i.e., the farm, landscape, and subnational regional scales (Cordingley et al., 2015; Karner et al., 2019; Karner et al., 2022). Most downscaling approaches for global SSPs are limited to quantitative, top-down methods, such as statistical downscaling, optimization, or spatial allocation procedures (e.g., Di Marco et al., 2019; Hoskins et al., 2016; Schipper et al., 2020). However, such procedures cannot adequately capture the complex farm-to-regional realities of land-use decision-making. Few examples exist where SSPs were coupled with BES assessments. For instance, Harrison et al. (2019) assessed impacts of SSPs for Europe (Kok et al., 2019) on land-use change, food production, carbon sequestration, water exploitation, flooding, and biodiversity (i.e., changes in species abundance) with integrated assessment models, yet still at a rather coarse resolution of approximately 16 km x 16 km. They found that socioeconomic factors drive greater land-use and food-production changes than climate change in Europe. In contrast, Neff et al. (2022) found that climate warming had a more significant impact on 40-year changes in insect diversity in Switzerland than land-use change. However, these downscaling approaches rarely involve the regional actors responsible for biodiversity-relevant decisions (with notable exceptions, e.g. Harrison et al., 2019; Nunez et al., 2020a). This creates a disconnect between scenario research in the context of BES and its practical application in environmental planning and management at the farm-to-regional scale. Yet, the literature shows that scenario processes involving regional stakeholders enhance ownership, credibility, relevance, and legitimacy (Garard and Kowarsch, 2017; Gramberger et al., 2015; Wiebe et al., 2018).

This study addresses the two key limitations by developing Agri-SSPs for four case study regions across four European countries with contrasting biophysical conditions and climate change impacts, applying a **novel protocol-driven participatory approach** that integrates quantitative and qualitative scenario elements **tailored to BES assessments**. We offer a **methodological advancement** that improves scenario usability, enhances stakeholder legitimacy, and extends the SSP framework toward actionable, biodiversity-relevant scenarios at regional scales consistent with the large-scale Eur-Agri-SSPs and comparable across each case study region. The scenario approach, hence, goes well beyond the definition of land-use drivers typical in participatory scenario processes. We define the following innovative scenario components: potential new/novel land-use and management practices (LUMPs) and land-use–biodiversity actions (LBAs), i.e., private and public actions triggering biodiversity-friendly land-use. Both components allow for capturing land-use choices (LUMPs) triggered by policy instruments and behavioral change mechanisms at the farm level (LBAs) — a gap typically neglected in larger-scale scenario applications. To our knowledge, this is the first presentation of a protocol-based approach for multi-regional scenarios that operationalizes the Eur-Agri-SSPs. The presented scenarios form the basis for subsequent BES assessments, which, however, are not part of this article.

## Methods and Data

In this section, the development of regional SSPs for four European case studies is presented. The regional SSPs are referred to as XX-Agri-SSPs in this manuscript (XX being an acronym for the respective case study region). The four case studies are the Lääne district (Läänemaa) in Estonia (LE), the Münsterland in Germany (ML), the Schwarzbubenland in Switzerland (SB), and the Wienerwald in Austria (WW). All case studies are at the subnational level with details included in supplementary materials (SM B). The following subsections first describe the conceptual framework (2.1) and, secondly, summarize the scenario development process (2.2), with full methodological details provided in the supplementary materials (SM A).

### Conceptual Framework

**Fig. 1:**
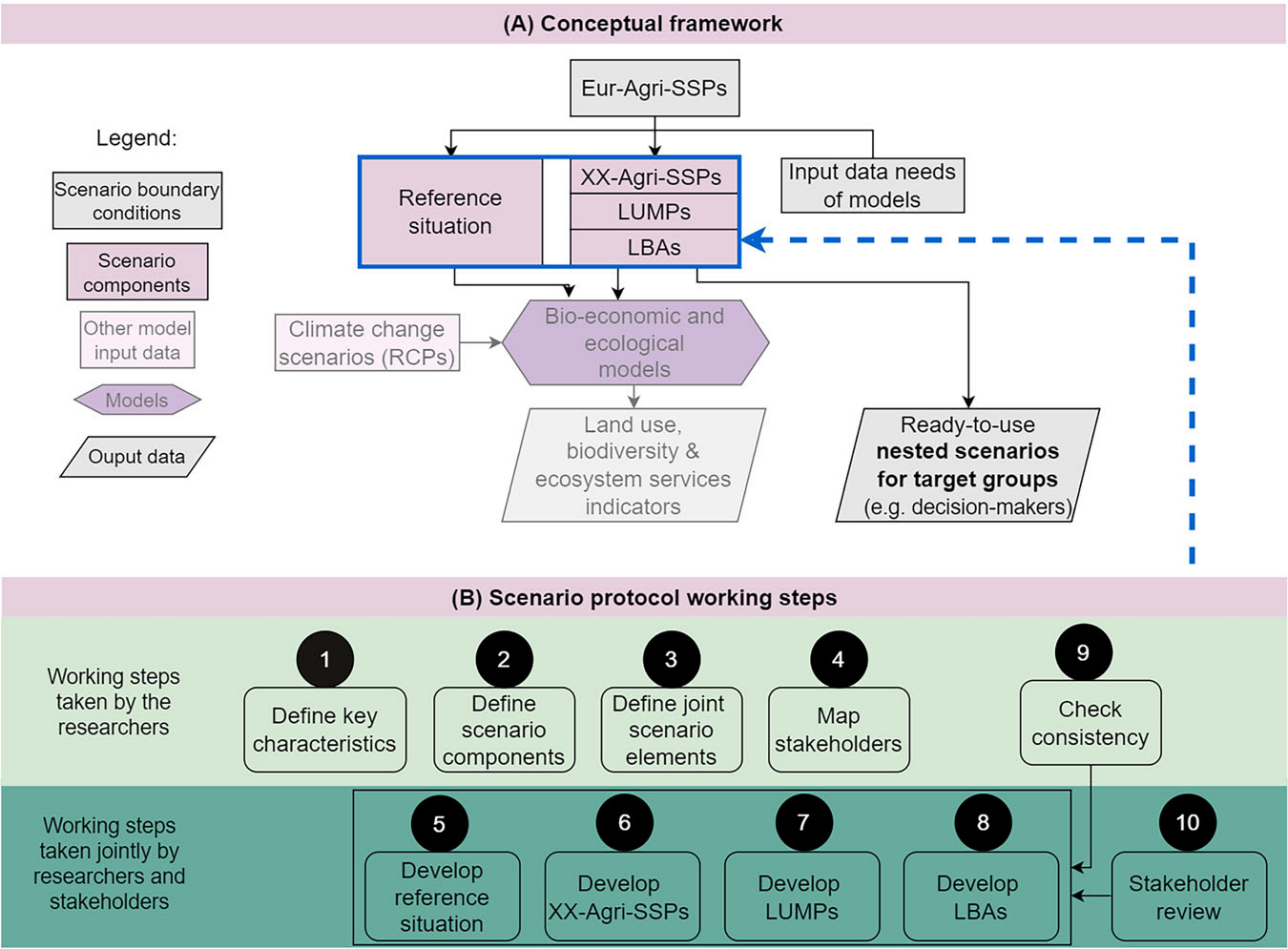
Overview of the conceptual framework **A** the scenario boundary conditions determine scenario components that form ready-to-use scenarios or model input data (not considered). The scenario components (blue box) are developed in a protocol-based scenario process with ten working steps **B**. It differentiates between working steps taken by the researchers only (light green) or jointly with stakeholders (dark green). Some working steps lead to feedback loops in the scenario development, as shown by the arrows. XX-Agri-SSPs refer to case study-specific Agri-SSPs, LUMPs are new land-use and management practices, and LBAs are land-use-biodiversity actions

The conceptual framework of this study is depicted at the top of Fig. 1. In its center are the scenarios for each case study, comprising the reference situation (REFobs) and future scenarios. XX-Agri-SSPs are vertically downscaled derivatives of the Eur-Agri-SSPs. They are complemented by new land-use and management practices (LUMPs) and land-use-biodiversity actions (LBAs) to form the future scenarios in this study (for details on these concepts, see section ``Working Step 2: Define Key Scenario Components”).

Two major boundary conditions determine the structure and expression (i.e., content) of the scenarios. First, we adopted a nested scenario approach to ensure vertical consistency across spatial scales. The Eur-Agri-SSPs provided the boundary conditions to develop corresponding XX-Agri-SSPs at the regional scale. Boundary conditions ensure that the main structure and expression of scenario elements (i.e., drivers) as well as the scenario logic are maintained at regional scales. Deviations from the trends in the Eur-Agri-SSPs are permissible, provided they do not impair the scenario logic (Zurek and Henrichs, 2007). For example, the pace of urbanization is increasing in Eur-Agri-SSP1 (Mitter et al., 2020) but this general European trend with its corresponding trend in rural marginalization may not hold for a specific region such as the WW, which is located adjacent to a metropolitan area prone to urban sprawl (Karner et al., 2024), thus facing an even faster pace of urbanization.

The second set of boundary conditions results from demand-side factors. Additional or more detailed scenario elements that complement the XX-Agri-SSPs, i.e., LUMPs and LBAs, were selected based on the input data requirements of integrated bio-economic models to assess BES, as well as on stakeholders’ information needs.

Similar to the Eur-Agri-SSPs, the system boundaries for the scenarios are defined by a broad and detailed specification of scenario elements that determine land-use decisions. However, the directions and levels of land-use change, and their corresponding effects on BES, are neither part of the scenario protocol nor determined in this article. Instead, the scenarios focus on the drivers of land-use change and BES change.

To develop the XX-Agri-SSPs, LUMPs, and LBAs, we adapted the protocol used to develop the Eur-Agri-SSPs (Mitter et al., 2019). It includes the regional downscaling and thematic extension of the Eur-Agri-SSPs as shown at the bottom of Fig. 1. The adapted protocol comprises ten iterative working steps, with feedback loops among some of them. Specifically, the consistency check and stakeholder review may lead to revisions to the scenarios. Five working steps are to be taken solely by the researchers, and five working steps engaged regional stakeholders.

### Scenario Protocol Working Steps

#### Working step 1: Define Key Characteristics and Analytical Scales

We identified a mismatch between the scale of existing scenario studies within SSP contexts, typical BES assessments, and decision-making regarding land-use and land-use policies (see section ``Introducation”). The primary goal of the scenarios in all case studies was to engage with and deliver results to regional stakeholders and to support quantitative assessments of BES. The main target groups were regional stakeholders with expected statistical and practical knowledge of the regional land-use characteristics that extend beyond single-field and farm management and can support the development of LUMPs and LBAs. The required knowledge of the land-use system determines the scale of the case study region. It is located between field, farm, and landscape scales, which are too small for land-use policy considerations, and may include BES applications and large-scale applications at the national level. Stakeholders included decision-makers (like mayors), regional managers (e.g., from the EU LEADER rural development program), farmers and other land managers (e.g., biosphere reserve managers), actors from education and extension services, as well as interest groups and NGOs from agriculture and BES in each case study. The thematic foci were the regional agricultural land-use systems with a special emphasis on drivers of regional agricultural land-use and BES. The temporal scale was the year 2050 in all case studies. This choice was determined by both the nested process that embedded the scenarios within a scenario hierarchy and the research interest.

#### Working Step 2: Define Key Scenario Components

The research consortium defined the scenario components in each region to ensure a shared understanding and communication of key terms between researchers and stakeholders. This step defined meta-information for each scenario component but did not specify its expression in any particular scenario; this is addressed in steps 6 to 8.

The XX-Agri-SSPs consist of two key components. A **narrative** is a qualitative description of the development of the scenario elements in the respective SSP. A **scenario element** is defined as a driver of the regional land-use system. We differentiated between socioeconomic drivers (like farm structure, market conditions, policies, and technology) and biophysical drivers (e.g., soil, slope). Climate change is not among the biophysical drivers; instead, it is complemented by climate change scenarios as described in working step 6.

The levels of a scenario element in each XX-Agri-SSP were specified both semi-quantitatively and quantitatively. The former is a verbal expression with terms like (medium or strong) increase/decrease for the time frame considered in the scenario. A quantitative development is expressed as a numeric relative or absolute change from a reference for the time frame considered in the scenario.

The XX-Agri-SSPs were complemented by LUMPs and LBAs to form the final scenarios. A **land-use and management practice (LUMP**) is a new land management/farming practice that has not been, or has been applied only rarely, in the region, with the potential to be applied in the future under a certain XX-Agri-SSP. Hence, a LUMP may include innovations not fully rolled out in the market (e.g., robots for weeding) or practices common in other regions but not yet applied in the case study. A detailed specification of LUMPs is required if models shall quantify the effects of the scenarios on BES.

**Land-use-biodiversity actions (LBA)** are private or public instruments that influence land-use decisions, including the selection of a particular LUMP, thereby enhancing the supply of BES. LBAs can be distinguished into three categories: information & institutions, legal standards, and economic instruments.

LUMPs and LBAs represent detailed specifications of selected scenario elements on top of an XX-Agri-SSP. Hence, different bundles of LUMPs and LBAs can be attributed to an XX-Agri-SSP as long as it remains consistent. LUMPs and LBAs reveal potential innovations in a region and complement the reference land-use system database to enable ex-ante BES, such as integrated models.

#### Working Step 3: Define Joint Scenario Elements

In the scenario context, drivers of agricultural land-use and BES are scenario elements in both the reference situation (REFobs, working step 5) and the XX-Agri-SSPs (working step 7). The research team defined a list of scenario elements to ensure comparability of scenarios and quantitative scenario results across case studies, using the Eur-Agri-SSPs as the initial set of scenario elements. We agreed on a small set of economic and political scenario elements that typically serve as quantitative inputs to bio-economic farm models. We deliberately limited the number of common scenario elements, as the participatory quantification of future developments is a time-intensive and complex task (see e.g. Karner et al., 2024). Each case study team was free to select additional scenario elements within their participatory processes.

#### Working Step 4: Map Stakeholders

Different stakeholder groups were involved in specific working steps of scenario development (for details, see SM A). We define stakeholders as individuals who are either affecting the regional land-use system in the case study, are affected by it, or both (Freeman et al., 2010). Each case study team was responsible for identifying relevant stakeholders and inviting them to engage in the participatory process, following the Prospex-CQI method (Gramberger et al., 2015). Here, criteria and categories for stakeholder identification are defined, followed by quotas for each category and the identification of individuals (see SM A). The consortium also defined interviews (see also working step 5) and two stakeholder workshops as the minimum level of stakeholder interaction for the scenario process.

#### Working Step 5: Develop Reference Situation

The observed reference situation (REFobs) is presented as a qualitative and quantitative description of the present regional agricultural land-use system. It is meant to (i) provide an agreed-upon, shared system boundary of the regional agricultural land-use system, (ii) inform the case study team about regional key issues related to land-use and BES, (iii) stimulate participation and stakeholder interactions, (iv) build a shared knowledge base and understanding on the regional land-use system and (v) provide a reference for scenario results. The design of REFobs was guided by a simple conceptual model of the agricultural land-use system, including drivers (such as climate, policies, prices, labor supply, or soils), major land-use categories (e.g., intensive or extensive cropland or grassland, forests, landscape elements, water bodies, infrastructure), major land-use effects in the past (i.e., related to BES), and land-use decision-making processes (i.e., the administrative level regarding land-use planning and the farm level). Semi-structured interviews (published in Suškevičs et al. 2023) and desk research using statistical databases, official documents, and geodata sets, as well as grey literature, were used to derive REFobs. The initial versions of REFobs were presented at the first stakeholder workshop and subsequently revised.

#### Working Step 6: Develop XX-Agri-SSPs

Each XX-Agri-SSP consists of two components: (i) (semi-)**quantitative developments** of the scenario elements, which are the basis for (ii) a scenario **narrative**. The Eur-Agri-SSPs describe average European developments and provide the boundary conditions. First, the consortium matched the region-specific scenario elements (working step 3) to those of the Eur-Agri-SSPs. Second, it selected scenario elements of the Eur-Agri-SSPs under strict boundary conditions at the regional scale, i.e., assuming that certain developments are given at the European level. Third, each case study team identified the scenario elements relevant to the regional context. These are the elements whose development is likely to deviate from the European average described by the Eur-Agri-SSPs, reflecting regional drivers and conditions. The preliminary list of scenario elements and their semi-quantitative developments were presented to stakeholders in stakeholder workshop 1 to validate REFobs and develop preliminary versions of the XX-Agri-SSPs. In general, workshop 1 followed the same structure across all case studies. As a starting point, the process was introduced, and results on identified climate-analogue regions were presented (Conradt, 2021). Climate-analogue regions today have climates comparable to those projected for each case-study region at a given time (e.g., 2050 or 2080), as identified using statistical distance methods (Mahony et al., 2017). After discussing the REFobs, the Eur-Agri-SSPs were presented in the plenary. Stakeholders discussed the different SSPs (for details and deviations in the workshop agenda across the cases, see supplementary material (SM) A). They were asked about the completeness and relevance of the list of scenario elements. Then, stakeholders discussed how the scenario elements, which are likely to develop differently in the case study, may develop in the discussed SSPs. Stakeholders were therefore asked to identify the development directions (i.e., directions of change) of these scenario elements using the provided background information. The case study teams then compiled the information from the workshop and prepared preliminary versions of the narratives.

The quantification of scenario elements and their development were based on literature review, modeling, and stakeholder and expert assumptions regarding agricultural input prices, output prices, and technology. Future yield levels were quantified with a statistical yield model (Conradt, 2022). Thus, the case study teams had to quantify developments for the farming system, farm structure, and policy measures within the scenario process. Each case study team selected a slightly different approach, driven by available time resources. The WW team used participatory quantification with fuzzy set theory for national-scale scenarios (the AT-Agri-SSPs; see Karner et al., 2024) and subsequently downscaling to the regional scale. The other case study teams, within their research groups, discussed how the scenario elements would develop quantitatively for each SSP, based on historical trends and expert assumptions.

#### Working Step 7: Develop LUMPs

Narrative validation and LUMPs specification for cropland and grassland were achieved in workshop 2. Stakeholders received the narratives for preparation before the workshop. First, the workshops began with a presentation of crop-yield changes, modeled under different climate scenarios (Conradt, 2022), to stimulate subsequent discussions on the LUMPs. The climate-analogue regions, previously presented in workshop 1, were briefly revisited to stimulate discussion of future crop alternatives. Second, the key features of each SSP were presented and discussed in the plenary. Third, LUMPs were identified and prioritized for each SSP. Discussions were structured along the three categories of the LUMPs (new crops, livestock and land-use; technology; and biodiversity measures). The case study teams compiled an initial list of LUMPs, incorporating discussions from workshop 1 and findings from literature reviews. Stakeholders were asked to (i) discuss which LUMPs are generally not relevant or redundant for the region, (ii) add missing LUMPs that would be relevant for the region, and (iii) prioritize and allocate the LUMPs to the different SSPs. Following workshop 2, the case study teams compiled the information, finalized the narratives, and selected LUMPs for each SSP.

#### Working Step 8: Develop LBAs

An initial list of **LBAs** was developed by the research consortium. Each case study team assessed the applicability in models, the expected impact on biodiversity, the case study’s priority, the relevance to each SSP, and the responsible governance level or institution, drawing on their expert judgment and the literature. Each criterion was assessed by the case study teams on a three-point scale to prioritize LBAs for workshop 2. In this workshop, LBAs were presented to stakeholders. Stakeholders could add missing LBAs, specify which LBAs were not relevant for the case study, and re-prioritize the LBAs.

#### Working Step 9: Check Consistency

Final XX-Agri-SSPs were developed through iterative consistency checks to increase vertical (external) and horizontal (internal) consistency (Mitter et al., 2019). Vertical consistency ensures that XX-Agri-SSPs align with their corresponding Eur-Agri-SSP. Horizontal consistency is achieved when all scenario elements within an individual XX-Agri-SSP are internally consistent. In addition, inter-scenario (scenario set) consistency ensures that differences between XX-Agri-SSPs are logically ordered and systematically reflect the intended contrasts between scenario narratives (e.g., stronger environmental ambition in SSP1 than in SSP2). Changes to the assumed development of individual scenario elements in an initially consistent scenario could require further adjustments to maintain consistency.

Horizontal and inter-scenario consistency were primarily checked by the case study teams, whereas vertical consistency across all case studies was assessed by a cross-case review team. This involved several rounds of communication with the case study teams and, where necessary, regional stakeholders. In general, XX-Agri-SSPs were considered vertically consistent when the quantitative change of a scenario element aligned with the corresponding semi-quantitative change (i.e., increase, decrease, constant/no change) defined in the Eur-Agri-SSPs. Limited deviations were accepted when justified by the regional context and valid assumptions. For example, while Eur-Agri-SSP1 assumes a constant pace of agricultural structural change, regional stakeholders and the case study team specified increasing numbers of farms in SB-Agri-SSP1, reflecting attractive financial compensation for orchard production—a widespread agricultural practice in the Schwarzbubenland. Additionally, the cross-case review team examined inter-scenario consistency across all case studies. For instance, consistency checks addressed cases in which scenarios with contrasting development logics (e.g., SSP1 vs. SSP2, or SSP2 vs. SSP5) showed only marginal differences in the development of scenario elements, prompting discussion of whether such limited divergence was plausible given their differing underlying assumptions.

#### Working Step 10: Stakeholder Review

In addition to consistency, the further quality criteria for scenarios are comprehensibility, plausibility, salience, legitimacy, richness of detail, and creativity, according to Mitter et al. (2019). The minimum standard for stakeholder reviews of the XX-Agri-SSPs required a discussion of the draft and final scenarios in workshops 1 and 2, respectively, with an emphasis on these quality criteria. Each case study team sent drafts (i.e., narratives and a table with semi-quantitative developments or quantitative data of scenario elements) to case study stakeholders for peer review before the second/third workshop. Additionally, the agenda and the aim of the workshop were sent to the stakeholders in time so that they were aware of the expectations for their roles and could prepare for the workshop discussions. Feedback from stakeholders was then incorporated by each case study team, potentially leading to changes to quantitative developments or narratives and, thus, requiring additional consistency checks.

## Results

This section presents summaries of the narratives of the XX-Agri-SSPs, quantitative scenario results, LUMPs, and LBAs. The full scenario dataset, including the observed reference situation (REFobs) and the case study XX-Agri-SSPs, is presented in the supplementary materials (SM B and SM C).

### Summaries of the XX-Agri-SSPs

Table 1 summarizes the commonalities and BES-specific developments in the scenario narratives of the XX-Agri-SSPs. It was designed to provide a quick overview of each XX-Agri-SSP to understand its inherent scenario logic, while presenting developments absent from European SSPs, such as the Eur-Agri-SSPs. General developments described here are in line with developments described in the Eur-Agri-SSPs. Differences between case studies are presented in the remaining results section. Table 1 was built using the narrative of each XX-Agri-SSP as input.

**Table 1:** Summaries of the narratives of the XX-Agri-SSPs

Topic	XX-Agri-SSP1 *Regional agriculture on sustainable paths*	XX-Agri-SSP2 *Regional agriculture on established paths*	XX-Agri-SSP5 *Regional agriculture on fossil-fueled, high-tech paths*
**Population and Urbanization**	**High social and environmental awareness**, attractiveness of smaller towns, **young and innovative farmers**, multifunctional landscapes	Population growth in urban agglomerations, **managed shrinkage** in rural areas, rising **awareness of environmental problems**, increasing agricultural education	Population growth only in urban areas, **high affinity for technology,** **detachment from nature**, belief in **technological substitution** of ecosystem services
**Economy**	**High demand for locally grown food**, local markets, **declining meat consumption,** **community-supported agriculture**, investments in training and technology lead to higher labor productivity	Stable price levels, gradual productivity increase, **rising land and water prices**, stable workforce due to **international immigration**	**Industrialization and global market integration**, high labor productivity, **low food prices**, abandonment of non-profitable land, rise of **corporate farms and indoor farming**
**Policies**	**Strict biodiversity** and resource-use **regulations**, high support for **agri-environmental-climate measures,** **result-based payments**, focus on **multifunctional land-use**, low, regionally differentiated income support	Medium income support, **area-based payments** remain, moderate support for rural development and **digitization,** **not sufficient** to reverse rural depopulation	Most subsidies **abandoned**, remaining support is via **financial instruments to ensure liquidity,** **relaxation of land-use restrictions**, nature and biodiversity **no longer valued**
**Technology**	Development of **environmentally friendly agricultural technologies**, measurement and marketing of environmental impacts, **public-private cooperation lead to high uptake**	Continued development of **precision farming** and **resource-efficient technologies**, better uptake due to improved education	**Strong focus on new technologies,** **technology valued in education,** **large-scale systems** like precision and controlled traffic farming dominate
**Natural Resources and Environment**	Continued recreational use of forests and landscapes, **dedicated recreation areas,** **restrictions on energy production on open spaces**, promotion of **agri-PV,** **reduced role** of biogas	**Intensified pressure on land**, increased competition (e.g., for solar/wind), biogas plays a **constant role**	**Reduction of environmental standards,** **technological solutions** for water supply and land amelioration, **segregation of recreational areas,** **slight increase in biogas** due to livestock growth

### Quantitative Developments of the XX-Agri-SSPs

**Fig. 2:**
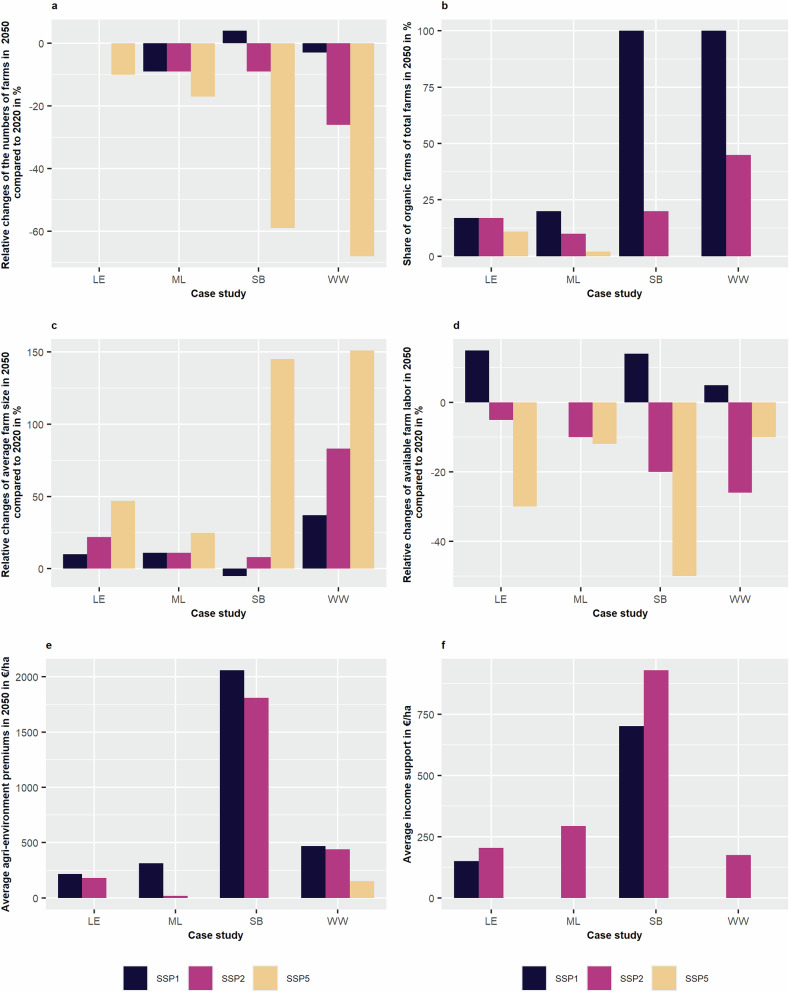
Relative changes compared to the reference situation REFobs or average data of six scenario elements in each SSP in 2050 (SSP1 in black, SSP2 in pink, and SSP5 in yellow) for each case study (LE – Läänemaa in Estonia, ML – Münsterland in Germany, SB – Schwarzbubenland in Switzerland, WW – Wienerwald in Austria): **a** changes of the total number of farms in the region, **b** share of organic farms of all farms in the region (shown because of very high relative changes), **c** changes of the average farm size in the region, **d** changes of available farm labor in the region, **e** the level of average agri-environment premiums in €/ha (shown because of very high relative changes in ML), **f** the level of income support for farmers in €/ha (for comparability with e). Please note: if no bar is shown, the respective value is zero

Figure 2 presents quantitative developments of farm structure and agricultural policies. Developments for farming systems, as well as all quantitative SSP data, are shown in SM C. The total number of farms decreases (Fig. 2a) in nearly all XX-Agri-SSPs and all case studies (except for LE-Agri-SSP1 and 2, SB-Agri-SSP1) compared to the reference situation in 2020. In particular, the number of farms changed between -9% (ML) and +4% (SB) in XX-Agri-SSP1, -26% (WW), and no change (LE) in XX-Agri-SSP2, and -68% (WW) and -10% (LE) in XX-Agri-SSP5.

Farm size increases in nearly all XX-Agri-SSPs except for SB-Agri-SSP1, where it declines (Fig. 2c). The average farm size increase is the largest in LE-Agri-SSP1 (84 ha), LE-Agri-SSP2 (93 ha), and LE-Agri-SSP5 (112 ha). Average farm size varies between 42 and 47 ha between the ML-Agri-SSPs, between 23 and 59 ha in SB-Agri-SSPs, and between 37 and 68 ha in WW-Agri-SSPs.

Figure 2b shows the share of organic farms among total farms in 2050. In XX-Agri-SSP1, the share of organic farms increases from 16% (LE), 2% (ML), 14% (SB), and 17% (WW) in REFobs to 17% (LE, +4%), 20% (ML, +836%), 100% (SB, +638%), and 100% (WW, +471%). In XX-Agri-SSP2, the share of organic farms ranges from 10% (ML) to 45% (WW). In SB/WW-Agri-SSP5, the share of organic farms declines to zero. In LE-Agri-SSP5, a relative reduction of 41% was specified, resulting in an 11% share of organic farms. In ML-Agri-SSP5, no change in the very low share of organic farms in 2020 was described.

Available farm labor (Fig. 2d) is expected to increase in XX-Agri-SSP1 in most case studies, varying between +5% to +15% compared to REFobs. In ML-Agri-SSP1, no change was specified. In SSP2 and SSP5, a decline was specified in all case studies. In LE, ML, and SB, the largest declines are specified for XX-Agri-SSP5, whereas in WW, they are specified for SSP2.

Nominal agri-environmental premiums, i.e., mean premiums per hectare, increase in two SSPs and all case studies compared to REFobs except for SSP5. The largest relative increases were specified for LE ( + 160% in SSP1, +116% in SSP2) and ML (16 times higher in SSP1) in general. However, these increases still result in lower absolute levels than those of SB and WW (Fig. 2e). Agri-environmental premiums increase by 25%/38% in SB/WW-Agri-SSP1, respectively, and by 10%/29% in SB/WW-Agri-SSP2, respectively. All case studies specified a phase-out of agri-environmental premiums in XX-Agri-SSP5, except for WW-Agri-SSP5, where stakeholders expected some premiums (150 €/ha, -56% compared to REFobs) due to its important role as a recreation area and for the provision of other ecosystem services for the Viennese population.

Income support for farmers is phased out in ML/WW-Agri-SSP1 and in all XX-Agri-SSP5 (Fig. 2f). Nominal income support declines by 25% in SB-Agri-SSP1 and increases by 33% in LE-Agri-SSP1, given the low overall financial support in LE in 2020. No changes were specified in ML/SB-Agri-SSP2, while an increase of 81% was specified for LE-Agri-SSP2 and a decline of 40% in WW-Agri-SSP2.

### LUMPs

LUMPs describe management measures that are new to the case study region, even if they are already common in other regions. LUMPs complement the bundle of regionally established management practices documented and described in the reference scenario REFobs. LUMPs include the adoption of novel crops, livestock, and land-use, as well as new technologies or measures to enhance BES. New crops specified as LUMPs include, for example, soy in all XX-Agri-SSPs (Table 2). New technologies include, among others, robots in LE/WW-Agri-SSP1 and SB-Agri-SSP2 and 5, agri-PV (photovoltaic systems) in ML/WW-Agri-SSP1, and conventional ground-mounted PV in ML/WW-Agri-SSP2. Agri-PV refers to photovoltaic plants that are installed in such a way that agricultural production is still possible. By contrast, ground-mounted PV refers to photovoltaic plants installed at ground level and that do not allow agricultural production. In ML, new technologies include slurry application, biogas plant expansion, and photovoltaic systems. New biodiversity measures include, e.g., 10% (5%, 1%) planting of landscape elements and additional greening measures per farm in ML-Agri-SSP1 (SSP2, SSP5) and in the respective WW-Agri-SSPs, or 3% in arable fields in SB-Agri-SSP1 and 2.

**Table 2:** Overview of the land-use and management practices (LUMPs) in each case study for each scenario

LUMPs	LE-Agri-SSPs	ML-Agri-SSPs	SB-Agri-SSPs	WW-Agri-SSP
**New crops, livestock, and land-use**				
New crops	Grain maize, chickpeas, lentils, winter barley, soy, sweet potato in all SSPs	Durum wheat, sunflower in all SSPs Soybean and other legumes in all SSPs Fruits and vegetables in SSP1: high share in SSP2: low share	Soy, maize, sunflower	Soy in all SSPs
New crops/breed varieties	ND	ND	In all SSPs (e.g. highly resistant varieties of soy)	e.g. stress/drought-resistant varieties of pumpkins and potatoes in all SSPs
Diversification of animals (e.g., ostrich, Angus cattle)	SSP2	ND	ND	SSP1, SSP2
Land consolidation	SSP5	ND	SSP5	SSP5
Conversion of land cover/use	ND	ND	Intercropping/ agroforestry in SSP5	Arable land in grassland in SSP1, SSP2
**New technologies**				
Tractors using biofuels or other alternative fuels	SSP1	ND	SSP1, SPS2	SSP2
Lightweight, small e-tractors, robots	SSP1, SSP5	ND	SSP2, SSP5	SSP1, SSP2
“Standard” Precision Farming (to maximize yield/ reduce fertilizer losses)	SSP1, SSP2, SSP5	ND	SSP5	SSP1, SSP2, SSP5
Biodiversity-friendly Precision Farming (focusing on spatially and temporally targeted management to minimize biodiversity impacts, e.g, optimized mowing dates, spatially differentiated inputs).	ND	ND	SSP1	SSP1
Photovoltaics (PV)	PV plants and wind parks in SSP1	Agri-PV (in strips between flowering): high share in SSP1, low share in SSP2 Ground-mounted PV: SSP2 and SSP5	ND	Agri-PV (bifacial) in SSP1; Ground-mounted PV and wind parks in SSP2; No PV in SSP5
Use of bio-compost, slurry separation (learning, developing methods)	SSP2	In all SSPs	SSP2	ND
Controlled traffic farming	SSP2	ND	SSP1	ND
Use of drones to protect wildlife etc.	ND	ND	SSP1, SSP2	SSP1, SSP2
**New biodiversity measures**				
A certain share of landscape elements per farm	Wider uncultivated grass margins to benefit birds and pollinators in SSP1 and SSP2	SSP1: 10%, SSP2: 5%, SSP5: 1%	3% Greening in arable fields in SSP1 and SSP2; 7% in total	SSP1: 10%, SSP2: 5%, SSP5: 1%
Rewilding	ND	ND	Of abandoned farm in SSP5	In nature protection areas in SSP1 and, SSP2, partly in abandoned farms in SSP5
Establishment of a network to support/maintain green infrastructure	SSP1	SSP1, SSP2	SSP1, SSP2	SSP1, SSP2

Note: Only those LUMPs are shown which have been discussed in at least two case studies; ND refers to not discussed in a case study. See SM B for the full list of LUMPs as defined in each case study.

### LBAs

Table 3 summarizes the most relevant LBAs and provides information on their general applicability in models, their expected impact on biodiversity, as assessed by the research team, their priority for each case study, their relevance for each SSP, and the required governance level or institution for introducing the LBA in a region. Table 3 shows only the three LBAs per category with the highest evaluation score. The evaluation score, presented in SM B, is calculated by the arithmetic mean of the three components: the applicability index, impact on biodiversity, and the average of the regional priority indices. SM B provides an overview of all mentioned LBAs and their respective evaluation scores. For instance, certification and labeling of high biodiversity value products were evaluated as highly relevant to LE/WW-Agri-SSP1 and LE/WW-Agri-SSP2, but difficult to represent in the models. An example of an LBA that was evaluated as relevant to the case studies and XX-Agri-SSPs due to its high impact on biodiversity, but appeared less applicable to the models, is raising consumers’ and producers’ awareness of their social and ecological responsibilities toward nature. Other high-ranked LBAs include civil engagement in landscape maintenance, guidelines for compiling landscape management plans at the farm level, and public championships among farmers for land-use actions, which may be used to raise awareness and increase marketing potential. Legal standards include, for instance, management standards, spatial planning, and long-term biodiversity monitoring, the latter of which is already established in Switzerland (Weber et al., 2004). Economic instruments, which can be implemented by governments and, to a lesser extent, by private companies or organizations, include, for instance, input and output taxes or subsidies for biodiversity-friendly production, higher crop and livestock diversity, and improved financial support for protected areas.

**Table 3:** Overview of the three highest-ranked(*) land-use - biodiversity actions (LBAs), their applicability in the models, expected impact on biodiversity, priority for each case study, relevance for the XX-Agri-SSPs, and associated governance levels/institutions

Category	LBA (Land-use - biodiversity action) description	Model applic-ability^1^	Impact on biodiv-ersity²	Priority for case studies²				Relevance for Agri-SSP²			Governance level or institution
				LE (EE)	ML (DE)	SB (CH)	WW (AT)	SSP1	SSP2	SSP5	
*Information & institutions*	Certification/labeling of products from heritage or high biodiversity farmland	2	2	3	2	already in place	3	3	3	1	State, industry
	Civil engagement in landscape maintenance	2	2	3	2	3	2	3	3	1	Subnational (provincial/ regional)
	Championships for land-use actions	2	3	3	ND	2	3	2	3	1	Subnational (provincial/ regional), industry
*Legal standards*	Management standards (e.g., input levels, schedules of fertilizers, pesticides, mowing)	2	3	2	2	3	2	3	2	1	State, industry
	Spatial planning: designing and designating ecological networks, valuable cultural landscapes, and valuable agricultural land	2	3	3	2	3	3	3	2	1	Subnational (provincial/ regional)
	Establish and finance a long-term biodiversity monitoring	2	3	3	2	already in place	3	3	2	1	State
*Economic instruments*	Implement result-based agri-environmental programs to preserve and enhance biodiversity (e.g., using target or indicator pollinator species).	2	3	3	2	2	3	3	3	1	State
	Subsidies for higher crop and livestock diversity, especially for rare and old varieties	2	3	2	2	2	2	3	3	1	State
	Improved financial support for protected areas and agri-environment-climate measures within agri-environmental programs	2	3	3	3	3	3	3	3	1	State

*A full table with all LBAs is included in Supplementary Material B

^1^ applicability: 1 - not possible, 2 - difficult, but possible, 3 - easily applicable

² priority/relevance: 1 - low, 2 - medium, 3 - high

*ND* = not discussed

Notes: the three highest-ranked actions per action group are shown (sum of applicability, impact, average priority, and average relevance), actions with higher applicability are prioritized if sums are equal

## Discussion

The discussion is structured into two parts. The first section compares scenario results across the four case studies and synthesizes general insights and policy implications beyond the level of individual case studies. The second section reflects on the methodology for nested participatory scenario design, is structured around six key challenges identified during scenario development, and provides corresponding recommendations.

### Differences and Commonalities Across Case Study Scenarios and Policy Implications

Our results reveal that European policy targets for organic farming—such as those articulated in the Farm-to-Fork strategy—are unlikely to be met uniformly across the case study regions even under the sustainability-oriented scenario XX-Agri-SSP1. This suggests that ambitious EU-level organic targets may overestimate the transformative capacity of market demand and agri-environmental payments in regions with unfavorable starting conditions, such as limited market maturity and weak value-chain development (e.g., ML), limited purchasing power, or already saturated organic markets (e.g., LE). This is somewhat in contrast to recent studies indicating that organic expansion can be substantial when market development and policy support align (Kremmydas, 2025; Möhring et al., 2024). Across our four case studies, organic farming expansion under XX-Agri-SSP1 is shaped by the interaction of consumer demand, policy incentives, and baseline land-use structures: regions with strong niche markets and favorable demand patterns can approach full conversion, whereas others show only modest increases due to structural or market constraints. This aligns with European land-use scenario studies showing that even under strong sustainability-oriented policies, only some normative visions are matched by plausible pathways, due to regional baseline conditions and path dependencies (Verkerk et al., 2018).

Differences in labor supply and farm structure further illustrate how the Eur-Agri-SSP1 scenario translates into divergent regional outcomes. Organic and biodiversity-friendly farming systems are generally associated with higher labor requirements (Bieniek-Majka and Guth, 2021; Crowder and Reganold, 2015). In XX-Agri-SSP1, higher labor demand is mostly met through an increased attractiveness of farming careers and a higher social status and valuation of farming. These findings align with Helfenstein et al. (2024), who emphasize that sustainable transformation requires an environment in which farmers are valued economically and socially. Yet, our study reveals that the ongoing structural shift toward fewer, larger farms continues to dominate long-term trajectories, meaning that the higher social status and attractiveness of farming in SSP1 are insufficient to counteract consolidation dynamics. This pattern is consistent with empirical evidence in LE, where rapid structural change has already occurred: farm numbers declined from ~56,000 in 2001 to ~11,000 in 2020, while average farm size increased, and the utilized agricultural area even increased (Rasva and Jürgenson, 2022; Statistics Estonia, 2020). Only in SB, higher attractiveness and social status – together with attractive financial compensation for orchard production – coincide with more stable farm numbers. Overall, these findings suggest that sustainability transitions tend to operate within ongoing structural change. Political objectives to stabilize farm numbers or rural employment typically require complementary structural and rural development policies.

The XX-Agri-SSP2 results across case studies indicate that the magnitude of agricultural change is driven less by overarching European policy ambitions than by national or subnational (including regional) implementation and its interaction with regional conditions. Specifically, public payments increase most in LE due to low 2020 baselines (European Commission, 2022) and national strategies to strengthen agri-environmental support (Regionaal- ja Põllumajandusministeerium, 2022). In WW, income support declines by 40%, while LE sees an 80% increase. The decline in WW is attributable to a shift in public payments from direct income support to agri-environmental premiums. ML and SB expect no major changes in income support.

XX-Agri-SSP5 outcomes further emphasize path dependencies, i.e., the importance of reference structural conditions in shaping agricultural futures. Regions with less consolidated agricultural structures experience accelerated farm exits (SB, WW) under this market- and technology-driven development pathway. Rega et al. (2019) confirm that such framework conditions might lead to large-scale abandonment of agricultural areas in Europe. Yet, according to our results, already consolidated systems show more limited additional change (LE). Regions with medium- to large-sized farm structures and intensive systems remain viable and relatively stable, where long-standing farming traditions and capital endowments prevail (ML). In most case studies, the share of organic farming decreases sharply under SSP5, reflecting strong price competition in globalized markets and the withdrawal of regulatory frameworks and public support. An exception is LE, where well-established organic markets and high sunk conversion investments create path dependencies that stabilize organic production despite an overall productivist trajectory. In WW, agri-environmental payments are not fully phased out, reflecting the region’s functional role in providing recreation and other ecosystem services to the Vienna metropolitan area. These patterns are consistent with findings from Helfenstein et al. (2024) showing that farm-level decision-making remains largely dominated by productivist principles, while multifunctionality tends to emerge primarily at the landscape level, mainly by regulatory necessity or little intensification opportunities - even under current framework conditions. This supports the interpretation that under SSP5-type futures, market-driven dynamics favor production-oriented systems unless multifunctional practices are either structurally locked in (as in LE) or supported by place-specific societal demand for ecosystem services (as in WW).

In the absence of regulatory frameworks and public support instruments, family farming becomes less attractive, particularly in regions with strong off-farm employment prospects in XX-Agri-SSP5 (e.g., Basel for SB and Vienna for WW). However, some part-time farms remain in these regions near cities. Although digitalization and high-tech agriculture increase productivity and attract some highly skilled, younger entrants, these developments do not offset the overall decline in family-operated farms. These developments stand in clear contrast to current EU policy objectives aimed at supporting family farms and promoting generational renewal, illustrating that such goals are often incompatible with market-driven pathways in which public support instruments and land-use regulations are largely absent under SSP5.

The key findings from the development of LUMPs and LBAs across different case studies emphasize the critical role of integrating innovative practices with biodiversity-promoting measures in shaping future agricultural land-use. For example, the potential adoption of soy production, as indicated by results from climate-analogue regions, suggests some homogenization of agricultural land-use across Europe, which can be mitigated by regionally tailored biodiversity measures. These results emphasize the need for region-specific, integrated strategies in which technological, policy, and market instruments work together to foster resilient agricultural systems.

### Challenges, Methodological Reflections, and Recommendations on Nested Participatory Scenario Development

**Fig. 3:**
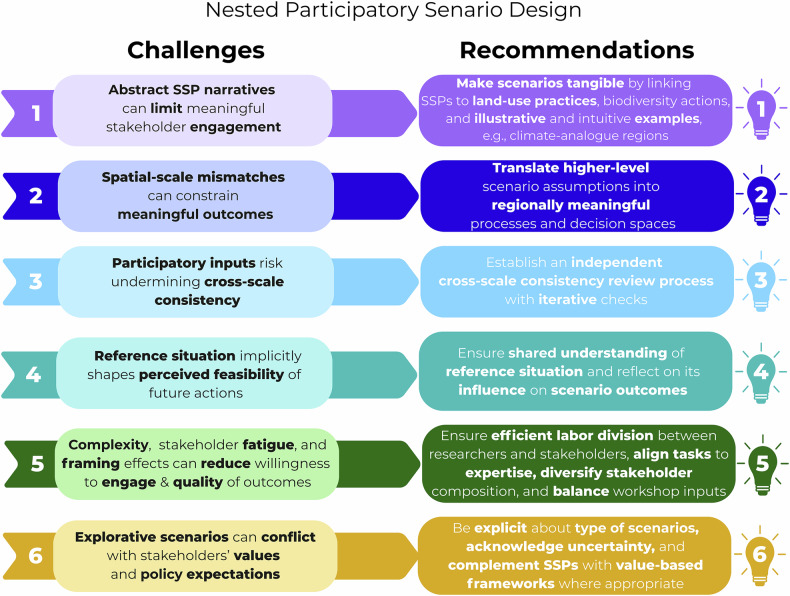
Overview of the identified six key challenges and corresponding recommendations for nested participatory scenario design

We now discuss the difficulties we encountered during scenario work by identifying six major challenges in nested participatory scenario design and by formulating recommendations to address them (Fig. 3).

#### Challenge 1: Abstract SSP Narratives Can Limit Meaningful Stakeholder Engagement

The integration of land-use and management practices (LUMPs) and land-use-biodiversity actions (LBAs) represents a key methodological advancement by making scenarios more tangible, actionable, and grounded in real-world decision-making contexts. LBAs also explicitly address the institutional compatibility of potential interventions, a dimension that has been highlighted as critical yet often underrepresented in scenario research (Hauck et al., 2019). Combined with a structured participatory scenario process, this approach enabled the development of region-specific pathways that are both consistent with the overarching Eur-Agri-SSPs and relevant for the planning and management of BES protection. Its applicability and usefulness for integrated modeling have been demonstrated through case studies in the Schwarzbubenland and Lääne County (Nishizawa et al., 2024, 2023, 2022).

Across the four case studies, stakeholders contributed to defining the development directions of scenario elements, specifying LUMPs, and reviewing and prioritizing LBAs. Their input was crucial for capturing regional specificities, including local governance settings, land-use histories, socioeconomic conditions, and the perceived attractiveness and feasibility of agricultural innovations. Consistent with previous research e.g., Tengö et al. (2017), place-based knowledge proved essential for identifying realistic LBAs and implementation barriers, thereby increasing the scenarios’ contextual relevance.

Stakeholders showed a stronger interest and greater confidence in discussing LUMPs and LBAs than in shaping rather conceptual socioeconomic scenario elements (challenge 1, Fig. 3). This may be due to their direct experience with farming practices and local policy instruments, which made these components more tangible and relevant. To support the engagement process, we presented results from an assessment of climate analogue regions (Mahony et al., 2017) at the first workshops. These climate analogues (Conradt, 2021)—which show the potential land-use future of each case study area by mirroring regions that currently face the expected climate conditions—proved particularly effective in stimulating discussions and improving participants’ understanding of potential changes. In the Austrian case study, this even prompted stakeholders to organize an excursion to the climate-analogue region of the Wienerwald to raise awareness among farmers and the public. Based on our experience from the cases, we recommend making scenarios tangible by linking SSPs to concrete aspects stakeholders can “anchor” themselves, e.g., land-use management practices, land-use-biodiversity actions, and illustrative and intuitive examples such as climate analogue regions (Fig. 3). Future scenario development processes could further strengthen this approach by extending the definition of LUMPs to also include management options that are expected to be reduced or phased out in individual scenarios, thereby capturing both transformative adoption and deliberate discontinuation pathways.

#### Challenge 2: Spatial-Scale Mismatches Can Constrain Meaningful Outcomes

Another challenge arose from mismatches between the spatial scale of scenario frameworks and stakeholders’ expertise. Across the case studies, challenges related to spatial scale highlight a broader issue in participatory scenario design: stakeholders’ expertise is typically anchored in regional or sector-specific contexts, whereas scenario frameworks (e.g., the SSPs) operate primarily at national or supranational scales. Such mismatches cannot be resolved solely through technical downscaling of variables but require explicit translation mechanisms that connect higher-level assumptions to regionally meaningful processes and decision spaces. There is a risk that when such translation is insufficient, stakeholder engagement becomes reactive rather than explorative and progressive. Multi-scale, possibly bottom-up, scenario-building approaches could be one way to address this (Kok et al. 2017). We found that structuring participatory processes to anchor discussions, after a thorough introduction of the SSP concept, first in land-use and management practices, then in land-use - biodiversity actions, i.e. policies, and subsequently linking them to higher-level socioeconomic assumptions, was very useful to break down the scenarios to the stakeholders’ regional realities and ensure constructive engagement (recommendation 2, Fig. 3). This may justify the partial switch of the process logic, which would rather suggest that higher level framework conditions determine regional land-use and policy reactions than vice versa. In short, we recommend translating higher-level scenario assumptions into regionally meaningful processes and decision spaces. Nevertheless, it is essential to align the study’s purpose, expected outcomes, and the competencies of the stakeholders involved with the spatial scale of the case study. Rather than prescribing an optimal case-study size, scale selection depends on factors such as farm structure, governance levels, knowledge systems, heterogeneity of farming systems, and bio-physical conditions, and often also on pragmatic considerations such as access to stakeholder networks. While the protocol presented here was developed for subnational regional scales with a focus on BES support, it can be adapted to larger or smaller spatial units with appropriate modifications.

#### Challenge 3: Participatory Inputs Risk Undermining Cross-scale Consistency

A key methodological insight concerns the interaction between participatory inputs and the need to maintain vertical (external), horizontal (internal), and inter-scenario consistency (challenge 3, Fig. 3). Iterative consistency checks conducted jointly by case study teams and a cross-case review team were essential to ensure that stakeholder-driven adaptations did not undermine alignment with the Eur-Agri-SSPs, internal consistency, or the intended contrasts between scenarios. Maintaining vertical consistency across multiple case studies proved particularly challenging, as it required balancing adherence to the overarching SSP framework with sufficient scope for regional differentiation when stakeholders demanded it. Maintaining stakeholders’ interest despite such constraint decision space appears to be a key focus.

Hence, we recommend establishing an independent cross-scale consistency review process with iterative checks and multiple communication rounds between a cross-case review team, case study teams, and stakeholders. Such an independent consistency review process proved indispensable in our case (recommendation 3, Fig. 3). While consistency checking itself is necessarily researcher-led due to its conceptual complexity across scales, communicating resulting revisions to stakeholders was important both for transparency and critical discussions, as well as for enriching the scenarios with additional arguments underpinning region-specific developments.

#### Challenge 4: Reference Situation Implicitly Shapes Perceived Feasibility of Future Actions

Stakeholders draw on different experiences and interpretations of their reference situation (REFobs)—including current biophysical conditions, status of biodiversity and ecosystem services, policy baselines, and land-use structures (challenge 4, Fig. 3). As a consequence, they may implicitly assess future developments against different starting points. This can impede the comparability of scenario outcomes within a region unless a shared reference situation is defined. The reference situation also appeared to shape stakeholder preferences for LUMPs and LBAs, as well as perceptions of feasible future actions. For example, regions with historically high agri-environmental payments or strong organic farming sectors tended to assume more ambitious biodiversity actions in SSP1 and SSP2. At the same time, stakeholders found it difficult to envision a technology-driven SSP5 future in these regions. In these cases, the case study teams were required to actively stimulate out-of-the-box thinking, for instance, by referring to technological developments that have already transformed these regions over the past, e.g., 50 years, and by highlighting emerging opportunities available today, such as virtual fencing. These observations reinforce the need to define and communicate the reference clearly, to explicitly reflect how baseline situations shape stakeholders’ preferences, and to contextualize scenario processes within regional realities rather than assuming uniform drivers across regions (recommendation 4, Fig. 3).

#### Challenge 5: Complexity, Stakeholder Fatigue, and Framing Effects Can Reduce the Willingness to Engage and the Quality of the Outcomes

The scenario development process was demanding for stakeholders both intellectually and in terms of time resources. It required contributions during workshops, interviews, and individual exchanges (challenge 5, Fig. 3). Stakeholders were invited to contribute to several complex tasks, including defining relevant drivers at the case study level, discussing development directions for regional scenario elements, participating in the quantification of selected variables, and co-designing LUMPs as well as LBAs. The well-documented problem of stakeholder fatigue (Gramberger et al., 2015) limited the theoretical engagement potential and necessitated pragmatic decisions. For example, the researchers decided to quantify certain scenario elements themselves, with subsequent stakeholder validation. In some cases, scientists’ familiarity with the regional agricultural systems, governance structures, and policy debates even enabled more grounded discussions, which may constrain the comparability across regions. However, this division of labor proved necessary and has been in line with recommendations from Karner et al. (2024), who suggest aligning quantified elements with the domain knowledge of participants (recommendation 5, Fig. 3). Stakeholder fatigue in participatory scenario processes tends to emerge from a combination of cognitive overload, normative tension, power asymmetries, limited perceived impact, and cumulative time and emotional burdens (e.g., Reed 2008; Gramberger et al. 2015; Mitter et al. 2019).

In addition to insufficient participation, the composition of stakeholder boards, including the range of attitudes, professional backgrounds, and experience levels, likely influenced the scenario content—particularly in the specification of LUMPs and LBAs. These components are inherently more subjective than the core SSP-aligned scenario elements, which were more tightly guided by the Eur-Agri-SSPs. Additionally, scientists’ role in structuring workshops and selecting workshop inputs can unintentionally frame discussions and bias the options considered. This challenge was addressed by deliberately presenting balanced information across crops, livestock and grassland-management, and technologies not yet prevalent in the region (e.g. virtual fences), alongside crop suitability and climate-analogue information (recommendation 5, Fig. 3). In summary, we recommend that future applications of the presented protocol should diversify stakeholder composition and transparently document framing choices and the considered alternatives, ensure efficient labor division between researchers and stakeholders, and align tasks to stakeholders’ expertise.

#### Challenge 6:Explorative Scenarios Can Conflict with Stakeholders’ Values and Policy Expectations

Finally, the explorative nature of the SSP scenarios posed challenges for engagement and trust-building between scientists and stakeholders (challenge 6, Fig. 3). Across the cases, we observed that trust in scenario processes emerged primarily from perceived procedural fairness and reflexivity rather than from methodological sophistication. Earlier literature suggests that credibility is built on transparency about the normativity of science, particularly in future studies (e.g., van der Hel 2018). The SSP framework encompasses a wide range of futures, from sustainability-oriented (SSP1) to those that pose significant challenges for climate change mitigation (SSP5). Some stakeholders struggled to engage meaningfully with SSP5 trajectories, particularly when they appeared to contradict current climate policy commitments at the regional, national, or EU levels, or when they did not align with stakeholders’ normative visions. In our cases, translating such scenarios into regional realities often required several rounds of discussion, supported by illustrative examples, to clarify uncertainty, the explorative nature of the scenarios, and their underlying assumptions (recommendation 6, Fig. 3).

These experiences suggest a need to further develop stakeholder-compatible scenario frameworks that allow for normative diversity without undermining credibility (recommendation 6, Fig. 3). One promising direction combines SSP-based narratives with alternative frameworks such as the Nature Futures Framework (NFF, Pereira et al., 2020), especially when co-developing sustainable or desired futures, while still retaining more critical, explorative trajectories for analytical completeness.

## Conclusions

This study developed and applied a protocol to develop nested scenarios, linking regional participatory scenarios to the Shared Socioeconomic Pathways (SSPs) by aligning them with the Eur-Agri-SSPs. A key innovation is the definition of new land-use and management practices (LUMPs) and land-use-biodiversity actions (LBAs). Another innovation is the simultaneous application of the protocol across multiple regional case studies, which ensures cross-scale and cross-case consistency and enhances policy relevance for supporting biodiversity and ecosystem services (BES) assessments and decision-making on land-use and land-use policies.

This coordinated approach enabled meaningful comparisons of scenario outcomes across European regions, highlighting the value of regional case work in complementing continental-scale assessments. While the Eur-Agri-SSPs offer single, generalized directions of change at the European level, our regionally grounded scenarios revealed both shared trends and important divergences. For example, scenario elements, shaped by existing funding schemes and socioeconomic contexts, vary substantially across scenarios and regions. Examples include the share of organic farms or payments for agri-environment-climate measures. From our findings, we draw the following general conclusions, which may be validated by further case studies conducted in other regions of Europe.

First, national and regional policy design and implementation have shown to be key leverage points that might critically mediate European ambitions. Divergent outcomes under similar European policy frameworks underscore that national and regional choices regarding the design, targeting, and sequencing of support instruments play a decisive role in shaping agricultural pathways. In this regard, translating EU-level sustainability targets to the regional level is critical, as regionally differentiated implementation pathways should enable scaling and sequencing of targets according to starting conditions, market capacity and maturity, and socioeconomic context.

Second, findings from SSP5 indicate that once market structures, investment patterns, and land-use systems are consolidated, policy leverage becomes limited. Early intervention and long-term policy stability are therefore crucial to avoid undesirable lock-ins and potentially irreversible losses of multifunctionality.

Third, as sustainability transitions unfold within complex rural systems, agri-environmental measures alone are unlikely to govern regional agricultural and rural outcomes such as changes in farm numbers or rural employment, highlighting the need for integrated policy mixes that combine environmental, structural, and rural development instruments.

Overall, this study offers a participatory methodology for downscaling global scenarios to the regional level in a way that is both engaging and applicable for stakeholders. By bridging global scenario logic with regional realities and incorporating innovative scenario components, the approach helps close the persistent gap between science, policy, and practice in regional planning and management to protect BES.

## Supplementary information

Supplementary information A

Supplementary information B

Supplementary information C

## Data Availability

Data is provided within the manuscript or supplementary information files.

## References

[CR69] Palazzo A, Vervoort MJ, Mason-D’Croz D, Rutting L, Havlík P, Islam S, Bayala J, Valin H, Kadi KAH, Thornton P, Zougmore R (2017) Linking regional stakeholder scenarios and sha red socioeconomic pathways: Quantified West African food and climate futures in a global context Global Environmental Change 45227−242. 10.1016/j.gloenvcha.2016.12.002 10.1016/j.gloenvcha.2016.12.002 PMC5637935 29056827

[CR1] Bieniek-Majka M, Guth M (2021) Factor Productivity And Profitability Of Horticultural Holdings In Selected Countries Specializing In Fruit And Vegetable Production In The European Union In The Period 2008-2018. Roczniki (Annals).

[CR2] Conradt T (2022) Choosing multiple linear regressions for weather-based crop yield prediction with ABSOLUT v1.2 applied to the districts of Germany. Int J Biometeorol 66:2287–2300. 10.1007/s00484-022-02356-5. 36056956 10.1007/s00484-022-02356-5 PMC9440329

[CR3] Conradt T (2021) SALBES Deliverable Report Deliverable 3.1 - Climate scenario report for the case study regions (No. 3.1 v2.0)

[CR4] Cordingley JE, Newton AC, Rose RJ, Clarke RT, Bullock JM (2015) Can landscape-scale approaches to conservation management resolve biodiversity–ecosystem service trade-offs? J Appl Ecol n/a-n/a. 10.1111/1365-2664.12545

[CR5] Crowder DW, Reganold JP (2015) Financial competitiveness of organic agriculture on a global scale. Proc Natl Acad Sci 112:7611–7616. 10.1073/pnas.1423674112. 26034271 10.1073/pnas.1423674112 PMC4475942

[CR6] Di Marco M, Harwood TD, Hoskins AJ, Ware C, Hill SLL, Ferrier S (2019) Projecting impacts of global climate and land-use scenarios on plant biodiversity using compositional-turnover modelling. Glob Change Biol 25:2763–2778. 10.1111/gcb.14663. 10.1111/gcb.14663 31009149

[CR7] Engström K, Olin S, Rounsevell MDA, Brogaard S, Van Vuuren DP, Alexander P, Murray-Rust D, Arneth A (2016) Assessing uncertainties in global cropland futures using a conditional probabilistic modelling framework. Earth Syst. Dynam. 7:893−15. 10.5194/esd-7-893-2016

[CR8] European Commission (2022) Environment and Climate Action (Summary) - Directorate-General for Agriculture and Rural Development [WWW Document]. URL https://agridata.ec.europa.eu/extensions/DashboardIndicators/Environment.html (accessed 10.22.24)

[CR9] European Commission (2020) Communication from the Commission to the European Parliament, the Council, the European Economic and Social Comittee and the Comittee of the regions. EU Biodiversity Strategy for 2030 - Bringing nature back into our lives (No. COM(2020) 380). Brussels

[CR10] Freeman RE, Harrison JS, Wicks AC, Parmar BL, Colle S de, 2010. Stakeholder Theory: The State of the Art. Cambridge University Press

[CR11] Garard J, Kowarsch M (2017) If at first you don’t succeed: Evaluating stakeholder engagement in global environmental assessments. Environ Sci Policy 77:235–243. 10.1016/j.envsci.2017.02.007.

[CR12] Gramberger M, Zellmer K, Kok K, Metzger MJ (2015) Stakeholder integrated research (STIR): a new approach tested in climate change adaptation research. Climatic Change 128:201–214. 10.1007/s10584-014-1225-x.

[CR13] Harrison PA, Dunford RW, Holman IP, Cojocaru G, Madsen MS, Chen P-Y, Pedde S, Sandars D (2019) Differences between low-end and high-end climate change impacts in Europe across multiple sectors. Reg Environ Change 19:695–709. 10.1007/s10113-018-1352-4.

[CR14] Helfenstein J, Hepner S, Kreuzer A, Achermann G, Williams T, Bürgi M, Debonne N, Dimopoulos T, Diogo V, Fjellstad W, Garcia-Martin M, Hernik J, Kizos T, Lausch A, Levers C, Liira J, Mohr F, Moreno G, Pazur R, Salata T, Schüpbach B, Swart R, Verburg PH, Zarina A, Herzog F (2024) Divergent agricultural development pathways across farm and landscape scales in Europe: Implications for sustainability and farmer satisfaction. Glob Environ Change 86: 102855. 10.1016/j.gloenvcha.2024.102855.

[CR15] Hoskins AJ, Bush A, Gilmore J, Harwood T, Hudson LN, Ware C, Williams KJ, Ferrier S (2016) Downscaling land-use data to provide global 30″ estimates of five land-use classes. Ecol Evolut 6:3040–3055. 10.1002/ece3.2104. 10.1002/ece3.2104 PMC4814442 27069595

[CR16] IPBES, 2019. Summary for policymakers of the global assessment report on biodiversity and ecosystem services of the Intergovernmental Science-Policy Platform on Biodiversity and Ecosystem Services. S Díaz, J Settele, ES Brondízio, HT Ngo, M Guèze, J Agard, A Arneth, P Balvanera, KA Brauman, SHM Butchart, KMA Chan, LA Garibaldi, K Ichii, J Liu, SM Subramanian, GF Midgley, P Miloslavich, Z Molnár, D Obura, A Pfaff, S Polasky, A Purvis, J Razzaque, B Reyers, R Roy Chowdhury, YJ Shin, IJ Visseren-Hamakers, KJ Willis, and CNZayas (eds.). IPBES secretariat, Bonn, Germany. 56

[CR17] Jaureguiberry P, Titeux N, Wiemers M, Bowler DE, Coscieme L, Golden AS, Guerra CA, Jacob U, Takahashi Y, Settele J, Díaz S, Molnár Z, Purvis A (2022) The direct drivers of recent global anthropogenic biodiversity loss. Sci Adv 8: eabm9982. 10.1126/sciadv.abm9982. 36351024 10.1126/sciadv.abm9982 PMC9645725

[CR18] Karner, K., Mitter, H., Schönhart, M., 2022. A Conceptualized Land Use System and Data to Support Integrated Landscape Assessments in Austria. pp. 211–227. 10.1007/978-3-658-36562-2_12

[CR19] Karner K, Mitter H, Sinabell F, Schönhart M (2024) Participatory development of Shared Socioeconomic Pathways for Austria’s agriculture and food systems. Land Use Policy 142: 107183. 10.1016/j.landusepol.2024.107183.

[CR68] Karner K, Cord FA, Hagemann N, Hernandez-Mora N, Holzkämper A, Jeangros B, Lienhoop N, Nitsch H, Rivas D, Schmid E, Schulp CJE, Strauch M, van der Zanden EH, Volk M, Willaarts B, Zarrineh N, Schönhart M (2019) Developing stakehold er-driven scenarios on land sharing and land sparing – Insights from five European case studies. J Environmental Management 241:488–500 10.1016/j.jenvman.2019.03.050 10.1016/j.jenvman.2019.03.050 30979560

[CR20] Keck F, Peller T, Alther R, Barouillet C, Blackman R, Capo E, Chonova T, Couton M, Fehlinger L, Kirschner D, Knüsel M, Muneret L, Oester R, Tapolczai K, Zhang H, Altermatt F (2025) The global human impact on biodiversity. Nature 641:395–400. 10.1038/s41586-025-08752-2. 40140566 10.1038/s41586-025-08752-2 PMC12058524

[CR21] Kok K, Bärlund I, Flörke M, Holman I, Gramberger M, Sendzimir J, Stuch B, Zellmer K (2015) European participatory scenario development: strengthening the link between stories and models. Climatic Change 128:187–200. 10.1007/s10584-014-1143-y.

[CR22] Kok K, Pedde S, Gramberger M, Harrison PA, Holman IP (2019) New European socio-economic scenarios for climate change research: operationalising concepts to extend the shared socio-economic pathways. Reg Environ Change 19:643–654. 10.1007/s10113-018-1400-0.

[CR23] Kok K, Rothman DS, Patel M (2006) Multi-scale narratives from an IA perspective: Part I. European and Mediterranean scenario development. Futures 38:261–284. 10.1016/j.futures.2005.07.001.

[CR24] Kok MTJ, Kok K, Peterson GD, Hill R, Agard J, Carpenter SR (2017) Biodiversity and ecosystem services require IPBES to take novel approach to scenarios. Sustain Sci 12:177–181. 10.1007/s11625-016-0354-8. 30174750 10.1007/s11625-016-0354-8 PMC6106191

[CR25] Kremmydas D, Beber C, Baldoni E, Ciaian P, Fellmann T, Gocht A, Hristov J, Pignotti D, Vicario DR, Stepanyan D, Tillie P (2025) The EU target for organic farming: Potential economic and environmental impacts of two alternative pathways. Appl Economic Perspect Policy 47:602–623. 10.1002/aepp.13470.

[CR26] Leclère D, Obersteiner M, Barrett M, Butchart SHM, Chaudhary A, De Palma A, DeClerck FAJ, Di Marco M, Doelman JC, Dürauer M, Freeman R, Harfoot M, Hasegawa T, Hellweg S, Hilbers JP, Hill SLL, Humpenöder F, Jennings N, Krisztin T, Mace GM, Ohashi H, Popp A, Purvis A, Schipper AM, Tabeau A, Valin H, van Meijl H, van Zeist W-J, Visconti P, Alkemade R, Almond R, Bunting G, Burgess ND, Cornell SE, Di Fulvio F, Ferrier S, Fritz S, Fujimori S, Grooten M, Harwood T, Havlík P, Herrero M, Hoskins AJ, Jung M, Kram T, Lotze-Campen H, Matsui T, Meyer C, Nel D, Newbold T, Schmidt-Traub G, Stehfest E, Strassburg BBN, van Vuuren DP, Ware C, Watson JEM, Wu W, Young L (2020) Bending the curve of terrestrial biodiversity needs an integrated strategy. Nature 585:551–556. 10.1038/s41586-020-2705-y. 32908312 10.1038/s41586-020-2705-y

[CR27] Mahony CR, Cannon AJ, Wang T, Aitken SN (2017) A closer look at novel climates: new methods and insights at continental to landscape scales. Glob Chang Biol 23:3934–3955. 10.1111/gcb.13645. 28145063 10.1111/gcb.13645

[CR28] Martins IS, Navarro LM, Pereira HM, Rosa IMD (2020) Alternative pathways to a sustainable future lead to contrasting biodiversity responses. Glob Ecol Conserv 22: e01028. 10.1016/j.gecco.2020.e01028.

[CR29] Meinshausen M, Smith SJ, Calvin K, Daniel JS, Kainuma MLT, Lamarque J-F, Matsumoto K, Montzka SA, Raper SCB, Riahi K, Thomson A, Velders GJM, van Vuuren DPP (2011) The RCP greenhouse gas concentrations and their extensions from 1765 to 2300. Climatic Change 109:213. 10.1007/s10584-011-0156-z.

[CR30] Mitter H, Techen A-K, Sinabell F, Helming K, Kok K, Priess JA, Schmid E, Bodirsky BL, Holman I, Lehtonen H, Leip A, Le Mouël C, Mathijs E, Mehdi B, Michetti M, Mittenzwei K, Mora O, Øygarden L, Reidsma P, Schaldach R, Schönhart M (2019) A protocol to develop Shared Socio-economic Pathways for European agriculture. J Environ Manag 252: 109701. 10.1016/j.jenvman.2019.109701. 10.1016/j.jenvman.2019.109701 31629178

[CR31] Mitter H, Techen A-K, Sinabell F, Helming K, Schmid E, Bodirsky BL, Holman I, Kok K, Lehtonen H, Leip A, Le Mouël C, Mathijs E, Mehdi B, Mittenzwei K, Mora O, Øistad K, Øygarden L, Priess JA, Reidsma P, Schaldach R, Schönhart M (2020) Shared Socio-economic Pathways for European agriculture and food systems: The Eur-Agri-SSPs. Glob Environ Change 65: 102159. 10.1016/j.gloenvcha.2020.102159. 32982074 10.1016/j.gloenvcha.2020.102159 PMC7501775

[CR32] Möhring N, Muller A, Schaub S (2024) Farmers’ adoption of organic agriculture—a systematic global literature review. Eur Rev Agric Econ 51:1012–1044. 10.1093/erae/jbae025.

[CR33] Mosnier A, Schmidt-Traub G, Obersteiner M, Jones S, Javalera-Rincon V, DeClerck F, Thomson M, Sperling F, Harrison P, Pérez-Guzmán K, McCord GC, Navarro-Garcia J, Marcos-Martinez R, Wu GC, Poncet J, Douzal C, Steinhauser J, Monjeau A, Frank F, Lehtonen H, Rämö J, Leach N, Gonzalez-Abraham CE, Ghosh RK, Jha C, Singh V, Bai Z, Jin X, Ma L, Strokov A, Potashnikov V, Orduña-Cabrera F, Neubauer R, Diaz M, Penescu L, Domínguez EA, Chavarro J, Pena A, Basnet S, Fetzer I, Baker J, Zerriffi H, Reyes Gallardo R, Bryan BA, Hadjikakou M, Lotze-Campen H, Stevanovic M, Smith A, Costa W, Habiburrachman AHF, Immanuel G, Selomane O, Daloz AS, Andrew R, van Oort B, Imanirareba D, Molla KG, Woldeyes FB, Soterroni, AC, Scarabello M, Ramos FM, Boer R, Winarni NL, Supriatna J, Low WS, Fan ACH, Naramabuye FX, Niyitanga F, Olguín M, Popp A, Rasche L, Godfray C, Hall JW, Grundy MJ, Wang X, 2022. How can diverse national food and land-use priorities be reconciled with global sustainability targets? Lessons from the FABLE initiative. Sustain Sci. 10.1007/s11625-022-01227-7

[CR34] Mouchet MA, Rega C, Lasseur R, Georges D, Paracchini M-L, Renaud J, Stürck J, Schulp CJE, Verburg PH, Verkerk PJ, Lavorel S (2017) Ecosystem service supply by European landscapes under alternative land-use and environmental policies. Int J Biodivers Sci, Ecosyst Serv Manag 13:342–354. 10.1080/21513732.2017.1381167.

[CR35] Nagesh P, Edelenbosch OY, Dekker SC, de Boer HJ, Mitter H, van Vuuren DP (2023) Extending shared socio-economic pathways for pesticide use in Europe: Pest-Agri-SSPs. J Environ Manag 342: 118078. 10.1016/j.jenvman.2023.118078. 10.1016/j.jenvman.2023.118078 37209644

[CR36] Neff F, Korner-Nievergelt F, Rey E, Albrecht M, Bollmann K, Cahenzli F, Chittaro Y, Gossner MM, Martínez-Núñez C, Meier ES, Monnerat C, Moretti M, Roth T, Herzog F, Knop E (2022) Different roles of concurring climate and regional land-use changes in past 40 years’ insect trends. Nat Commun 13: 7611. 10.1038/s41467-022-35223-3. 36509742 10.1038/s41467-022-35223-3 PMC9744861

[CR37] Nishizawa T, Kay S, Schuler J, Klein N, Conradt T, Mielewczik M, Herzog F, Aurbacher J, Zander P (2023) Towards diverse agricultural land uses: socio-ecological implications of European agricultural pathways for a Swiss orchard region. Reg Environ Change 23:97. 10.1007/s10113-023-02092-5. 37489177 10.1007/s10113-023-02092-5 PMC10363045

[CR38] Nishizawa T, Kay S, Schuler J, Klein N, Herzog F, Aurbacher J, Zander P (2022) Ecological–Economic Modelling of Traditional Agroforestry to Promote Farmland Biodiversity with Cost-Effective Payments. Sustainability 14:5615. 10.3390/su14095615.

[CR39] Nishizawa T, Schuler J, Bethwell C, Glemnitz M, Semm M, Suškevičs M, Hämäläinen L, Sepp K, Värnik R, Uthes S, Aurbacher J, Zander P (2024) Modelling Alternative Economic Incentive Schemes for Semi-Natural Grassland Conservation in Estonia. Environ Manag 74:757–774. 10.1007/s00267-024-02011-2. 10.1007/s00267-024-02011-2 PMC11393159 39090440

[CR40] Nunez S, Alkemade R, Kok K, Leemans R (2020) Potential biodiversity change in Central Asian grasslands: scenarios for the impact of climate and land-use change. Reg Environ Change 20:39. 10.1007/s10113-020-01619-4.

[CR41] O’Neill BC, Carter TR, Ebi K, Harrison PA, Kemp-Benedict E, Kok K, Kriegler E, Preston BL, Riahi K, Sillmann J, van Ruijven BJ, van Vuuren D, Carlisle D, Conde C, Fuglestvedt J, Green C, Hasegawa T, Leininger J, Monteith S, Pichs-Madruga R (2020) Achievements and needs for the climate change scenario framework. Nat Clim Chang 10:1074–1084. 10.1038/s41558-020-00952-0. 33262808 10.1038/s41558-020-00952-0 PMC7688299

[CR42] O’Neill BC, Kriegler E, Ebi KL, Kemp-Benedict E, Riahi K, Rothman DS, van Ruijven BJ, van Vuuren DP, Birkmann J, Kok K, Levy M, Solecki W (2017) The roads ahead: Narratives for shared socioeconomic pathways describing world futures in the 21st century. Glob Environ Change 42:169–180. 10.1016/j.gloenvcha.2015.01.004.

[CR70] Tschumi P, Seidl I, Pütz M, Gubler L (2026) Advancing national Shared socioeconomic pathways (SSPs): A novel procedure applied to develop current Swiss SSPs Global Environmental Change 96:103105. 10.1016/j.gloenvcha.2025.103105.

[CR43] Pedde S, Kok K, Onigkeit J, Brown C, Holman I, Harrison PA (2019) Bridging uncertainty concepts across narratives and simulations in environmental scenarios. Reg Environ Change 19:655–666. 10.1007/s10113-018-1338-2.

[CR44] Pereira LM, Davies KK, den Belder E, Ferrier S, Karlsson-Vinkhuyzen S, Kim H, Kuiper JJ, Okayasu S, Palomo MG, Pereira HM, Peterson G, Sathyapalan J, Schoolenberg M, Alkemade R, Carvalho Ribeiro S, Greenaway A, Hauck J, King N, Lazarova T, Ravera F, Chettri N, Cheung WWL, Hendriks RJJ, Kolomytsev G, Leadley P, Metzger J-P, Ninan KN, Pichs R, Popp A, Rondinini C, Rosa I, van Vuuren D, Lundquist CJ (2020) Developing multiscale and integrative nature–people scenarios using the Nature Futures Framework. People Nat 2:1172–1195. 10.1002/pan3.10146.

[CR45] Powers RP, Jetz W (2019) Global habitat loss and extinction risk of terrestrial vertebrates under future land-use-change scenarios. Nat Clim Chang 9:323–329. 10.1038/s41558-019-0406-z.

[CR46] Priess JA, Hauck J, Haines-Young R, Alkemade R, Mandryk M, Veerkamp C, Gyorgyi B, Dunford R, Berry P, Harrison P, Dick J, Keune H, Kok M, Kopperoinen L, Lazarova T, Maes J, Pataki G, Preda E, Schleyer C, Görg C, Vadineanu A, Zulian G (2018) New EU-scale environmental scenarios until 2050 – Scenario process and initial scenario applications. Ecosyst Serv, SI: Synthesizing OpenNESS 29:542–551. 10.1016/j.ecoser.2017.08.006.

[CR47] Rabin SS, Alexander P, Henry R, Anthoni P, Pugh TAM, Rounsevell M, Arneth A (2020) Impacts of future agricultural change on ecosystem service indicators. Earth Syst Dyn 11:357–376. 10.5194/esd-11-357-2020.

[CR49] Rasva M, Jürgenson E (2022) Agricultural Land Concentration in Estonia and Its Containment Possibilities. Land 11:2270. 10.3390/land11122270.

[CR50] Reed MS (2008) Stakeholder participation for environmental management: A literature review. Biol Conserv 141:2417–2431. 10.1016/j.biocon.2008.07.014.

[CR51] Rega C, Helming J, Paracchini ML (2019) Environmentalism and localism in agricultural and land-use policies can maintain food production while supporting biodiversity. Findings from simulations of contrasting scenarios in the EU. Land Use Policy 87: 103986. 10.1016/j.landusepol.2019.05.005.

[CR52] Regionaal- ja Põllumajandusministeerium, 2022. Euroopa Liidu ühise põllumajanduspoliitika strateegiakava 2023–2027 [Estonian Common Agricultural Polcy Strategic Plan 2023-2027] [WWW Document]. URL https://www.agri.ee/euroopa-liidu-uhise-pollumajanduspoliitika-strateegiakava-2023-2027 (accessed 10.22.24).

[CR53] Rosa IMD, Purvis A, Alkemade R, Chaplin-Kramer R, Ferrier S, Guerra CA, Hurtt G, Kim H, Leadley P, Martins IS, Popp A, Schipper AM, van Vuuren D, Pereira HM (2020) Challenges in producing policy-relevant global scenarios of biodiversity and ecosystem services. Glob Ecol Conserv 22: e00886. 10.1016/j.gecco.2019.e00886.

[CR54] Schipper AM, Hilbers JP, Meijer JR, Antão LH, Benítez-López A, de Jonge MMJ, Leemans LH, Scheper E, Alkemade R, Doelman JC, Mylius S, Stehfest E, van Vuuren DP, van Zeist W-J, Huijbregts MAJ (2020) Projecting terrestrial biodiversity intactness with GLOBIO 4. Glob Change Biol 26:760–771. 10.1111/gcb.14848. 10.1111/gcb.14848 PMC7028079 31680366

[CR56] Statistics Estonia, 2020. Agricultural Census 2001, 2020

[CR57] Suškevičs M, Karner K, Bethwell C, Danzinger F, Kay S, Nishizawa T, Schuler J, Sepp K, Värnik R, Glemnitz M, Semm M, Umstätter C, Conradt T, Herzog F, Klein N, Wrbka T, Zander P, Schönhart M (2023) Stakeholder perceptions of agricultural landscape services, biodiversity, and drivers of change in four European case studies. Ecosyst Serv 64: 101563. 10.1016/j.ecoser.2023.101563.

[CR58] Tengö M, Hill R, Malmer P, Raymond CM, Spierenburg M, Danielsen F, Elmqvist T, Folke C (2017) Weaving knowledge systems in IPBES, CBD and beyond—lessons learned for sustainability. Curr Opin Environ Sustain Open Issue, Part II 26–27:17–25. 10.1016/j.cosust.2016.12.005.

[CR59] van der Hejden K (2005). Scenarios: The Art of Strategic Conversation, 2nd Edition. ed. Wiley

[CR60] van der Hel S (2018) Science for change: A survey on the normative and political dimensions of global sustainability research. Glob Environ Change 52:248–258. 10.1016/j.gloenvcha.2018.07.005.

[CR61] van Vuuren DP, Edmonds J, Kainuma M, Riahi K, Thomson A, Hibbard K, Hurtt GC, Kram T, Krey V, Lamarque J-F, Masui T, Meinshausen M, Nakicenovic N, Smith SJ, Rose SK (2011) The representative concentration pathways: an overview. Climatic Change 109:5. 10.1007/s10584-011-0148-z.

[CR62] Verkerk PJ, Lindner M, Pérez-Soba M, Paterson JS, Helming J, Verburg PH, Kuemmerle T, Lotze-Campen H, Moiseyev A, Müller D, Popp A, Schulp CJE, Stürck J, Tabeau A, Wolfslehner B, van der Zanden EH (2018) Identifying pathways to visions of future land use in Europe. Reg Environ Change 18:817–830. 10.1007/s10113-016-1055-7.

[CR63] Walker WE, Harremoës P, Rotmans J, Van Der Sluijs JP, Van Asselt MBA, Janssen P (2003) Defining Uncertainty: A Conceptual Basis for Uncertainty Management in Model-Based Decision Support. Integr Assess 4:5–17. 10.1076/iaij.4.1.5.16466.

[CR64] Weber D, Hintermann U, Zangger A (2004) Scale and trends in species richness: considerations for monitoring biological diversity for political purposes. Glob Ecol Biogeogr 13:97–104. 10.1111/j.1466-882X.2004.00078.x.

[CR65] Wiebe K, Zurek M, Lord S, Brzezina N, Gabrielyan G, Libertini J, Loch A, Thapa-Parajuli R, Vervoort J, Westhoek H (2018) Scenario Development and Foresight Analysis: Exploring Options to Inform Choices. Annu Rev Environ Resour 43:545–570. 10.1146/annurev-environ-102017-030109.

[CR66] Zerriffi H, Reyes R, Maloney A, 2022. Pathways to sustainable land use and food systems in Canada. Sustain Sci. 10.1007/s11625-022-01213-z 10.1007/s11625-022-01213-z PMC9575642 36275780

[CR67] Zurek MB, Henrichs T (2007) Linking scenarios across geographical scales in international environmental assessments. Technol Forecast Soc Change 74:1282–1295. 10.1016/j.techfore.2006.11.005.

