# Supplementary material for: Participatory Scenario Design to Support Ex-ante Biodiversity and Ecosystem Services Assessments in Four European Agricultural Case Studies
Source: Environ Manage. 2026 Apr 1;76(4):135. doi: 10.1007/s00267-026-02435-y (PMC13043572; doi:10.1007/s00267-026-02435-y)
Supplement: Supplementary file 1 — Supplementary information A [file 267_2026_2435_MOESM1_ESM.docx]

**Supplementary Material A for the submitted manuscript “Participatory scenario design to support ex-ante biodiversity and ecosystem services assessments in four European case studies”**

The supplementary material A contains details on the method for stakeholder identification and interaction (A.1) and on how the workshops varied in each case study in working step 7 in order to develop the XX-Agri-SSPs (A.2).

## A.1 Working step 4: Stakeholder identification, grouping and define interaction

**Case study stakeholders** were involved in the participatory scenario process in specific steps. Each case study team was responsible for identifying relevant case study stakeholders and inviting them for the participatory process. To identify relevant stakeholders, the case study teams tried to identify key stakeholders with their professional networks and internet-based searches on key organizations (such as regional chambers, organizations etc.). Then, the case study team used the snowball technique to identify all relevant stakeholders. Each case study team aimed for at least 10 participating stakeholders in each stakeholder interaction. The SALBES consortium defined that all possible views and values should be integrated in the scenario process and defined a list of minimum stakeholder groups that should be invited (i.e. farmers and farmer representatives, nature conservation and agricultural administration, landscape planners/regional governments, ministries (environment, agriculture), scientists and NGOs focusing on either agricultural land use, ecosystem services or biodiversity).

Each case study team was responsible for identifying relevant stakeholders and inviting them to the participatory process by following the Prospex-CQI method (Gramberger et al., 2015). First, “C” – criteria and categories had to be defined for identifying stakeholder groups. The main criterion was, that a diversity of views was integrated. The identified relevant categories were the organization of the stakeholders (i.e. farmers and farmer representatives, administration, policy, science/education, and NGOs) and the thematic focus of the organization (agriculture, land use, and BES). Second, the “Q” – quota had to be set for all categories. This was defined such that at least one representative of each thematic focus should be included for each organization, resulting in at least ten stakeholders. Third, “I” – individuals that fit the categories were identified. The case study teams tried to identify key stakeholders with their professional networks and internet-based searches on organizations. Then, they used the snowball technique (Parker et al., 2019) to identify additional relevant stakeholders. Table A1 below shows details about each stakeholder interaction in each case study, related to the respective aims of the interaction.

Table A1: Overview of the stakeholder interactions in the scenario development process in each case study (LE (EST)- Läänemaa in Estonia, ML (GER) – Münsterland in Germany, SB (CH) – Schwarzbubenland in Switzerland and WW (AT) – Wienerwald in Austria)

| Stakeholder interaction (approx. duration) | Aims | Date | | | |
| --- | --- | --- | --- | --- | --- |
|  |  | **LE (EST)** | **ML (GER)** | **SB (CH)** | **WW (AT)** |
| Interviews  (30-120 minutes) | - Develop REFobs | 01/2020 – 06/2020 | 01/2020 – 03/2020 | 01/2020-03/2020 | 01/2020 – 03/2020 |
| Workshop 1  (2-4 hours) | - Validate REFobs, - Identify important regional drivers and - their development in each scenario - Identify LUMPs | 12/2020 | 03/2020 | 01/2021 | 04/2021 |
| Workshop 2  (2-4 hours) | - Validate future scenarios, - (Identify and) Prioritize LUMPs and - Identify LBAs | 06/2022 | 12/2022  (including WS 3) | 04/2022 | 05/2022 |
| Workshop 3  (2-4 hours) | - Review and validate model assumptions, first model results and scenarios, - Identify and prioritize LBAs | - | 12/2022 | - | 01/ 2023 |

## A.2 Deviations in working steps 5 to 8 in the individual working steps

The SALBES consortium defined the minimum required stakeholder interaction, i.e. interviews and three stakeholder workshops for the scenario process. The stakeholder interaction is described in more detail in the respective sub-sections of the SALBES scenario methodology section in the main manuscript. In the LE case, some bilateral e-mail and phone exchanges were also held additionally, foremost with the MoRural Affairs representatives (e.g. in terms of LUMPs) and they have also consulted a few experts outside their immediate research team on e.g. agronomy, soil science, and organic agriculture. Certain data to fill in the SoP was compiled on requests by PRIA (Agricultural Registers and Information Board in Estonia). Details regarding the stakeholder workshops in each case study are presented next.

Workshop 1

In Austria, the case study team preselected a list of scenario elements which are likely to develop differently in the Wienerwald compared to the Austrian average as specified in the AT-Agri-SSPs and which are of particular relevance in the region, as identified with REFobs and the interviews. During the workshop, the case study team first presented short summaries of the AT-Agri-SSPs and the preselected scenario elements. Then, three breakout groups were defined, where the development of those scenario elements were discussed for one of the three selected SSPs (SSP1, SSP2 or SSP5) by case study stakeholders. Additionally, case study stakeholders were asked to identify plausible LUMPs for each scenario, differentiated for cropland, extensive (1- or 2-cut) and intensive grassland and landscape elements.

In CH, the first workshop was help online in January 2021. Herein, the study team gave an overview of the SALBES project as well as highlights from the stakeholder interviews. Afterwards, first results of a regional inventory regarding economic and ecological farm and farmland indicators were presented. Furthermore, current and future climate scenarios (temperature and precipitation) were shown and discussed with a special focus on climate similarity regions. Afterwards the concept and the main principles of SSPs were introduced and the special characteristics of REF, SSP1, SSP2 and SSP5 were shown. As a group we discuss each SSP1s in the context of the Swiss study region and collect specifics for each SSP for modelling purposes. The focus of the discussion was on modern and traditional orchards as typical element in the region.

In EST, first workshop (held virtually on 6th of December 2020), nine stakeholders besides our project team were present: 1 MoE and 1 MoRural Affairs’ representative, 2 farmers, 1 agricultural extension specialist, 1 scientist, 1 regional government representative, 1 nature conservation admin, 1 regional NGO (LEADER action group). We presented the general info about the SALBES project and then drafts of REFobs and SSP1, SSP2 and SSP5. In the discussions in breakout groups, we focused on drivers of changes and stakeholders’ ideas about what elements could be added to each of the three scenario descriptions. We have taken the received input into account in revising the REFobs and the scenario summaries. We shared the group discussion summaries with the stakeholders after the workshop via e-mail.

The first SALBES workshop in Münsterland, DE on 10.03.2020 had the following participants: 3 farmers, 1 representative of the regional government, 1 representative of the organic farming association, 2 representatives of different farmer unions, 1 representative of the chamber of agriculture, 6 scientists, 2 representatives of a nature conservation association, 3 representatives of the land management foundation, 1 representative of the regional farming newspaper and a moderator. The workshop started with an overview of the project. The participants were then presented with the first results from stakeholder interviews and some desktop research. These results focused on the perception of climate change, economic development, politics and institutions, technology, and the environment. Then, the stakeholders were presented with the results of regional climate change modeling. This information provided valuable insights into the potential impacts of climate change on the region. The workshop also included an input session where participants discussed what should change in the case studies and what should stay the same. This discussion aimed to reflect on the concept of a safe operating space on a regional level. Regarding the scenario work, a presentation of draft scenarios and REFobs was already given, followed by a discussion with the stakeholders. The discussion allowed for an in-depth exploration of the scenarios and their implications. A summary of the discussion was also provided.

Workshop 2

In AT, the case study team presented first project results regarding modelled climate change impacts on crop yields for the selected climate scenarios and empirical results on the past development of some biodiversity indicators. Then, the case study team presented the short summaries of the WW-Agri-SSPs and the LUMPs. Case study stakeholders then prioritized the LUMPs for the WW-Agri-SSP2 (in total 23 LUMPs were presented) to reduce the number for the subsequent modelling. Then, case study stakeholders were asked to identify likely impacts of the LUMPs and the WW-Agri-SSP2 on regional land use, ESS and biodiversity – which should be used on the one hand for validation purposes of the subsequent modelling and for the definition of LBAs. Finally, case study stakeholders were asked to identify possible LBAs to enhance the expected status of biodiversity and ESS in the respective WW-Agri-SSP. Due to time constraints, only WW-Agri-SSP2 could be discussed in this workshop. The case study team asked the case study stakeholders to contribute to the discussion of the other two WW-Agri-SSPs, their impact on land use, ESS and biodiversity and the LBAs via a shared online document.

In CH, the focus of the second workshop was on modelling results and discussion of LUMPs and LBAs. In the first part, the study team presented results of the climate modelling. The development of crop yields for 2030, 2050 and 2080 considering different climate scenarios as well as a shortlist of “future” crops (e.g. corn maize, sunflower, soya) with high yield potential in future climate scenarios were presented. Afterwards, economic and ecological modelling results were shown. As in the first workshop the focus was on modern (=tree planting optimized for machinery use) and traditional orchards (i.e. scattered trees often requiring manual management and harvesting) and the interaction between biodiversity promotion and farm economics. The second part of the workshop focused on the discussion with and between stakeholders regarding the future development of the region in light of the SSPs. Three drawings (for each SSP one landscape image) were given to the stakeholders. Based on that, the group discussed the pictures and structured their results by topics such as development of arable, fruit, and livestock production, farmland technology options, market developments and biodiversity promotion. For each SSP, LBAs were defined in line with the discussed sub-topics. Since time was short, the final list of LBAs were sent out by email and feedback was received for some specific management options such as irrigation and swarm robot use.

In EST, the second workshop was attended by 5 stakeholders outside of our team: 2 agricultural admin, 2 MoRural Affairs and 1 regional nature conservation administration representative. It should be mentioned that at least 2 of these stakeholders were also at the same time part-time farmers in Lääne County. In this workshop, we briefly revisited the three SSPs and presented climate and crop yield modelling results (T. Conradt), as well as some ideas for future technologies (M. Mielewczik). Due to time constraints, we focused the workshop on Land Use- Biodiversity Actions drafts (prepared by our team) for each of the three SSPs and not the LUMPs. This may mean some limitations, but in the process of drafting LUMPs and LBAs in our team, we realised that the LUMPs are reflected in the LBAs well, and we did a cross-check (in a table format) to make sure each LUMP corresponded to at least one LBA. At the second workshop, besides seeking ideas on additional LBAs, we have asked the stakeholders to comment on and prioritize the pre-drafted LBAs: questions about the applicability; potential impact on biodiversity and general priority for the region. We have received constructive feedback on the LBA drafts and some additional ideas from the stakeholders. In total together with the stakeholders, we defined 18 LBAs for SSP1, 8 LBAs for SSP2 and 11 LBAs for SSP5 (there was some minimal overlap with certain LBAs, i.e. they were relevant to multiple SSPs). We shared the updated LBAs’ descriptions with the workshop participants after the workshop via e-mail.

The second workshop in Münsterland, DE, took place on Dec. 7, 2022. Participants were: 3 farmers, 1 representative of the regional government, 1 representative of the regional government, 1 representative of an organic farming association, 4 representatives of the farmer union with different expertise (livestock, environment, photovoltaics), 6 scientists, 2 representatives of a nature conservation association, 3 representatives of the land management foundation. After a short reminder on the aims of the project, the participants were presented with the status of scenario development. The presentation highlighted the goals and methodology of the project. To stimulate discussion, results from the Swiss case study were shared. The workshop then further went into the contents of the scenarios, focusing on technologies, agri-environmental measures, and new policy instruments. New technologies, such as photovoltaics and N-mitigation/livestock farming/biogas plants, were discussed within the context of the Agri-SSPs. Agri-environmental measures, including new biodiversity measures were also explored. Measures on arable land were: 1. annual and perennial flowering mixtures, each with and without biogas use, 2. extensive cereal cultivation, 3. pole bean-maize mixture, 4. maize cultivation with clover/grass undersowing, 5. rapeseed with double row spacing (allowing mechanical weeding instead of pesticides). Measures on grassland comprised: 1. Extension of horse keeping (more pastures, late mowing on meadows), 2. Extension of sucker cow systems, 3. Strips of older grass patches. The following measures were suggested as measures for supporting “Green Infrastructure”: 1. Establishment of a network to support/maintain green infrastructure and 2. a certain share of landscape elements per farm.

The discussion concluded with an examination of new policy instruments (see Table SM.B 5.5: Overview of the LBAs for Münsterland in Germany) that could support the implementation of the scenarios and facilitate the transition to more sustainable farming practices. The workshop ended with a summary of the key points and an outlook for future steps and potential areas of further collaboration.

Workshop 3

The main aims of the third workshop were to review and validate model assumptions, first model results and scenarios and to identify and prioritize LBAs. A third workshop was just held in the WW, AT during January 2022 in the case study region. As modelling was not as advanced as planned, only very preliminary results were presented in WW. In contrast, modelling was fast advancing in SB, which is why the aims of the third workshop were already covered in the second workshop. Hence, a third workshop was not needed to be held in SB. In ML and LE, time constraints and in particular shared modelling capacities did not allow to start the modelling on time to present any results in a stakeholder activity. The German modelling team also conducted the modelling for the case study region in SB, with which they started first, and LE. Hence, modelling was somewhat delayed for ML and LE. Therefore, no third workshop was held in both case studies. Yet, some modelling assumptions were discussed in the second workshop.
