# Supplementary material for: Participatory Scenario Design to Support Ex-ante Biodiversity and Ecosystem Services Assessments in Four European Agricultural Case Studies
Source: Environ Manage. 2026 Apr 1;76(4):135. doi: 10.1007/s00267-026-02435-y (PMC13043572; doi:10.1007/s00267-026-02435-y)
Supplement: Supplementary file 2 — Supplementary information B [file 267_2026_2435_MOESM2_ESM.docx]

**Participatory scenario design to support ex-ante biodiversity and ecosystem services assessments in four European agricultural case studies**

# **Supplementary Material B** –

**Observed reference situation and XX-Agri-SSPs for each case study for the submitted manuscript “Participatory scenario design for ex-ante biodiversity and ecosystem services assessments in four European case studies”**

This supplementary material B contains results of the stakeholder mapping, case study descriptions and the observed reference situation (REFobs), the scenarios, consisting of the narratives for the XX-Agri-SSPs, the LUMPs and the LBAs, for each case study. Quantitative developments for the joint scenario elements are shown in supplementary material C.

Table of Content

[Supplementary Material B – 1](#_Toc203173631)

[B.1 Stakeholder mapping 3](#_Toc203173632)

[B.2 Observed reference situation (REFobs) 4](#_Toc203173633)

[Short case study descriptions 5](#_Toc203173634)

[Reference situation for Läänemaa 7](#_Toc203173635)

[Reference situation for Münsterland 8](#_Toc203173636)

[Reference situation for Schwarzbubenland 9](#_Toc203173637)

[Reference situation for Wienerwald 9](#_Toc203173638)

[B.3 Narratives 10](#_Toc203173639)

[XX-Agri-SSP1: Regional agriculture on sustainable paths 10](#_Toc203173640)

[XX-Agri-SSP2: Regional agriculture on established paths 11](#_Toc203173641)

[XX-Agri-SSP5: Regional agriculture on fossil-fueled, high-tech paths 12](#_Toc203173642)

[Läänemaa 13](#_Toc203173643)

[Münsterland 15](#_Toc203173644)

[Schwarzbubenland 16](#_Toc203173645)

[Wienerwald 18](#_Toc203173646)

[B.4 LUMPs 20](#_Toc203173647)

[Läänemaa 20](#_Toc203173648)

[Münsterland 21](#_Toc203173649)

[Schwarzbubenland 22](#_Toc203173650)

[Wienerwald 23](#_Toc203173651)

[B.5 LBAs 24](#_Toc203173652)

[References 34](#_Toc203173653)

## B.1 Stakeholder mapping

The following table B 1.1 shows summary results of the involved stakeholders in the scenario development in each case study. Exact organizations and names are not provided due to data protection reasons. The selected criteria for the stakeholder mapping were that the following organizations are integrated: farmers and farmer representatives, administration, policy, science and NGOs). Furthermore, the thematic focus of the organization (agriculture, land use and biodiversity and ecosystem services, BES) was identified as a criterion for stakeholder selection. Only stakeholders who participated in at least one workshop are include in the table below. Suškevičs et al. (2023) present a mapping of the interviewed stakeholders.

| Organization | Thematic focus | Läänemaa (Estonia) | | Münsterland (Germany) | | Schwarzbuben-land (Switzerland) | | Wienerwald (Austria) | |
| --- | --- | --- | --- | --- | --- | --- | --- | --- | --- |
|  |  | Female | Male | Female | Male | Female | Male | Female | Male |
| Farmer/ represent-tatives | Agri-culture | 1 | 2 | 2 | 7 | 2 | 2 | 1 | 4 |
|  | BES |  |  |  |  | 1 |  | 2 | 2 |
| Admin-istration | Agri-culture | 1 | 1 | 1 | 1 | 1 | 1 | 2 | 1 |
|  | BES | 1 |  |  | 2 | 1 |  | 2 | 1 |
| Policy | Agri-culture | 2 |  |  | 1 |  |  | 0 | 1 |
|  | BES | 1 |  | 1 |  |  |  | 0 | 0 |
| Science/  Education | Agri-culture |  |  |  | 1 |  | 1 | 1 | 0 |
|  | BES | 1 |  |  | 1 | 1 | 1 | 0 | 1 |
| NGO | Agri-culture |  |  |  | 1 |  |  | 1 | 0 |
|  | BES |  |  |  | 1 |  | 3 | 5 | 3 |
| ***All*** | ***All*** | ***7*** | ***3*** | ***4*** | ***15*** | ***6*** | ***8*** | ***14*** | ***13*** |

## B.2 Observed reference situation (REFobs)

In the following, first a summary table for the reference situation of each case study is provided, second short case study descriptions and finally a translated and shortened version of the reference situation of each case study is presented.

Table B.2: Data for the the reference scenario REFobs in year 2020 for each of the four case studies for joint scenario elements, data sources are listed in Supplementary Material C

| Category | Parameter | Unit | LE, EE | ML, DE | SB, CH | WW, AT |
| --- | --- | --- | --- | --- | --- | --- |
| Biophysical constraint | Average field size | ha | 6 | 4.06 | <1 | 1.12 |
| Farm structure | Total farms | Number | 489 | 10235 | 74 | 1237 |
|  | Farm size | ha/farm | 76 | 37.7 | 24.1 | 27.2 |
|  | Farm labor availability | hrs/year per farm | 3883 | 2955 | NA | 5400 |
|  | Participation in agri-environmental program | Share of farms/farmland | 39.1% of farmland | 34% of farms | NA | 52% of farmland |
|  | Organic farms | Share of total farms | 16% | 1.94% | 14% | 17% |
| Input prices | Fuel | €/l * | 0.951 | 0.89 | 1.051 | 1.112 |
|  | Feed | €/kg * | NA | 0.26^1^ |  | 0.24 |
|  | Labor | €/AWU * | 18655 | 18509 | 74709^2^ | 26415^3^ |
| Policy measures** | Average agri-environmental premium | €/ha of UAA* | 83 | 18 | 1644 | 340 |
|  | Income support premium | €/ha of UAA* | 113 | 294 | 934 | 175 |
|  | Nature protection contract premiums | €/ UAA ha* | 64 | 275-685 (grassland)  70–980 (additional measures grassland)  25-1980 arable land | 374 | 700 (on grassland) / 900 (on cropland) |
|  | Premiums for Areas with Natural Constraints | €/ha UAA* | NA | 35-115 (100% for farms until 80 ha, 80 % for farms between 80-120 ha, 0 % for farms >120 ha) | 280 | 50 |

Notes: UAA = utilized agricultural area, AWU = agricultural working unit
* CHF have been converted to EUR using the exchange rate of 0.9341 €/CHF for 2020 (European central bank 2023; <https://www.ecb.europa.eu/stats/policy_and_exchange_rates/euro_reference_exchange_rates/html/eurofxref-graph-chf.en.html> )
** Actual premium flow in each case study region, average across all farms of one case study. In ML, nature protection contract premiums and premiums for Areas with Natural Constraints are officially set ranges of the premiums and not the average across actual premium flows.
^1^ feed in ML is based on the prices of barley (188.1€/t) and soy (330 €/t)
^2^ median salaries of a Swiss full-time job in the whole economy (private and public sector)
^3^ for skilled labor (with education)

### Short case study descriptions

Lääne County (LE) is one of Estonia's 15 counties, situated in Western Estonia and spanning an area of 1816 km². The county is organized into three municipalities: one urban municipality - Haapsalu and 2 rural municipalities Vormsi and Lääne-Nigula. The topography of Läänemaa is predominantly flat. Positioned entirely within the lowlands of West Estonia, Lääne County boasts a coastline stretching approximately 415 km. Notably, 23% of its territory is dedicated to nature protection, including the Matsalu National Park. Agriculture dominates with extensive use, small fields, meadows and small forest patches. The region features remarkable biological diversity, surpassing many other areas in Estonia. However, insufficient grazing or mowing within extensive agricultural systems, combined with land abandonment, has led to declines in species abundances, shifts in species composition, and alterations in landscape structure.

The Münsterland region (ML) (6752 km²) is located in north-west Germany (Federal State of North Rhine-Westphalia) and comprises five counties as well as the city of Münster. This region features a small-structured agricultural landscape also known as “Münsterland Park-like landscapes”, with open agriculturally used land, i.e. arable fields and grassland patches, and with characteristic landscape elements, i.e. small wooded patches, hedges and small water bodies. Arable land for intensive livestock production and, to a lesser extent, for cash crop production dominates the land use. The region is also known as an “energy region” for its high production of renewable energy by photovoltaic systems, wind power and biogas plants.

The Schwarzbubenland region (SB) covers an area of 42.2 km² in north-western Switzerland (SB), in the canton of Solothurn and has been listed in the federal inventory of “Landscapes and Natural Monuments of National Importance” since 1983. The hilly region (between 300 m - 735 m) hosts a beautiful landscape scenery consisting of a mosaic of orchards, cropland, grassland, forests, and small villages. The development of the region and its inhabitants was and is still strongly influenced by the nearby metropolis of Basel.

The Austrian Wienerwald region (WW) extends across an area of 1050 km² and has been designated a biosphere reserve since 2005. In total 58 municipalities comprise the biosphere reserve, seven of which belong to the city of Vienna and the others to the province of Lower Austria. The area is characterized by a hilly terrain on a sea-level from 200 m up to 890 m. Its landscape is characterized by a mosaic of forests, grasslands, vineyards and cropland.

Figure 1 provides an overview of the location of each case study in Europe, in the context of their bio-geographical region and observed and projected climate change.


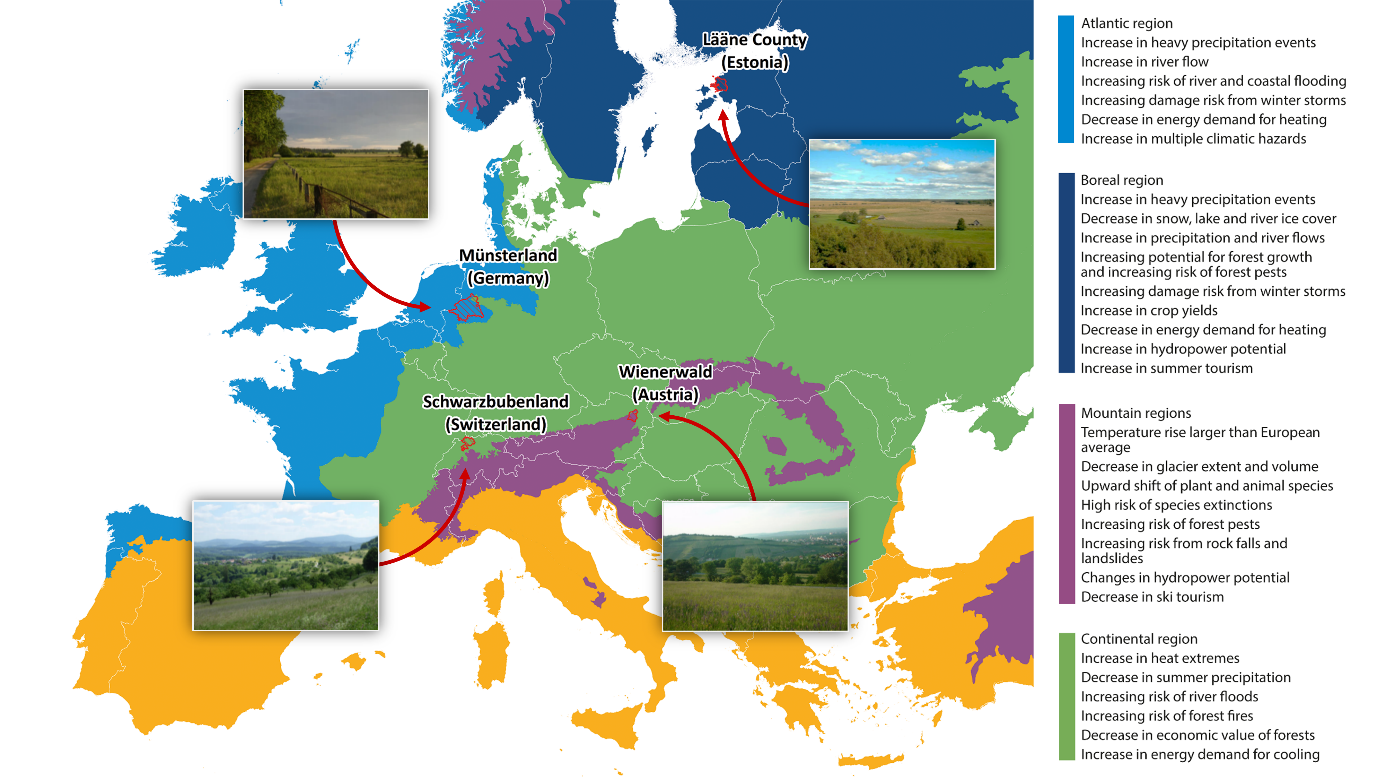
Figure 1: Case studies’ locations in relation to the bio-geographical European region and key observed and projected climate change impacts (adapted from European Environment Agency, 2017)

Figure 2 shows the share of land cover classes in each case study. LE and WW are dominated by forests (54% and 70%, respectively), while ML is dominated by arable land (55%). The share of permanent grassland varies between 12% (WW) and 20% (LE).


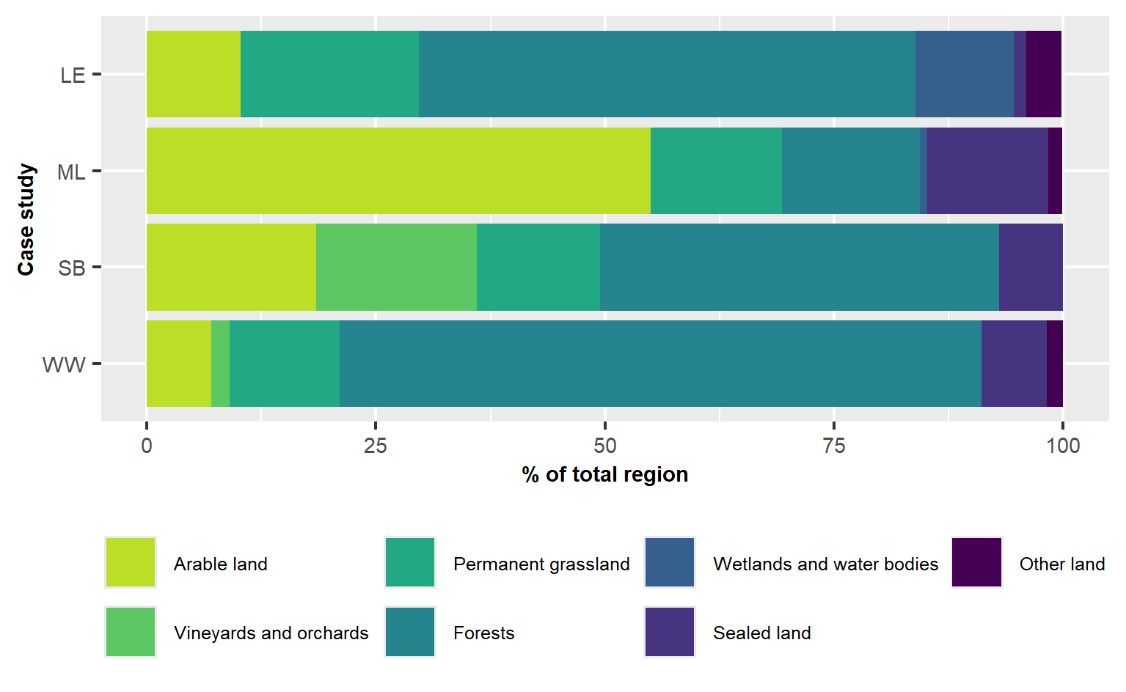


Figure 2: Share of land cover in each case study (WW- Wienerwald in Austria, SB-Schwarzbubenland in Switzerland, ML- Münsterland in Germany and LE-Läänemaa in Estonia)

### Reference situation for Läänemaa

Lääne County (*Läänemaa* in Estonian), one of the 15 counties in Estonia is situated in the West Estonia at the coast of Baltic Sea. It lies about 100 km from the capital Tallinn and consists of 3 municipalities: Vormsi, Lääne-Nigula municipalities and Haapsalu town. The county covers an area of 1816 km2 (181 557 ha), with a population of 20507 inhabitants (2019). The population trend during last decades has been negative.

The whole county’s landscape is predominantly flat, as it is situated entirely on the West-Estonian Lowland. The average annual temperature is between 6.1 °C and 7.8 °C with a precipitation of 500 to 700 mm. The past climate trends over the last 60 years show a clearly visible and statistically significant upward trend in temperatures. The precipitation average for the period 1961–2020 was 656 mm/a with a standard deviation between single years of 105 mm which corresponds to a coefficient of variation of 16.0%. However, the annual precipitation values also expose a less significant increasing trend (p< 1.3· 10−3). Reduced precipitation is observed in spring, which is a regional phenomenon, even more pronounced in the previous climate period.

Agricultural land covers ca. 20% of the county’s territory, including 11% of arable land and 9% of permanent grassland (PRIA, 2020). Forest covers 53% (96 370 ha) and yards 1% of the county surface (2020, Land cadastre statistics). The remaining significant part of the county’s territory is categorized as natural grassland or “other land” - mainly semi-natural grasslands with insufficient or unclear management. The total area of protected areas (land territory and water area protected under the Nature Conservation Act) makes up 29% of the total area of Lääne County (2020, Estonian Environment Agency) incl. the most famous Matsalu National Park.

Constraints to agricultural activity include physical constraints, foremost soil quality (e.g. clayish or stony soils and peaty areas): arable land and natural grassland productivity score/weighted average real bonitet is 33 (the average in Estonia is 40). The share of peat soils in the total area of agricultural land in Läänemaa is 19% on the basis of the existing soil map and PRIA register (average value in Estonia is 10.7%). Constraints are also set by land use planning (e.g. permanent grasslands, valuable landscapes) or other structural conditions, e.g. market conditions, land availability (all suitable fields are already in the hands of big farmers).

In 2020, Läänemaa had 489 farms (info from the main subsidies’ system database, PRIA). In 2018, the average farm in Lääne County was 73 ha and had an average yearly revenue of 43 thousand euros. By 2020, the average farm size had increased to 76 ha. Average size of the managed field was 6 ha (2020). Whereas the average farm did not increase in terms of agricultural land when comparing 2006 and 2018, average revenue has almost doubled during this period. From 2014 onwards, beef cattle is the dominant livestock animal in Läänemaa, at least when compared to cows, sheep and goats. Number of cows and sheep has not changed substantially, whereas the number of goats has decreased by more than half. There are farms growing lamas, chinchillas, alpacas, crayfishes, carps, burgundy snails, silver foxes, bees. There are also more unconventional birds in poultry as peacock, ostrich, pigeon and pheasant. Many farms mix livestock. Diversification outside agriculture can be estimated by share of non-farm income. While this share fluctuated between 16% and 4% in 2014-2015, from 2016-2018 it was around 10%. Thus, on average 10% of income for farmers in Läänemaa is earned outside farming.

Prices of inputs and outputs of farm production between 2006 and 2018 have been generally increasing in Läänemaa. Highest increase has been in wages. Meanwhile, prices of livestock output have also been increasing. The economic analysis of Lääne County carried out by the Lääne County Foundation in 2015-2019 showed that the number of agricultural holdings has increased, but the number of employees has decreased. The main reason is production automation. The increase in wages in Lääne County, as elsewhere in Estonia, has also depended on the annual increase in the minimum wage, which ranges from 6.4% to 10.9% in 2013-2018. If we look at the salaries of Lääne County in the same position compared to the average salaries in Estonia, the salaries of Lääne County employees are about 20% lower. The agricultural census in 2020 clearly showed that agricultural production is concentrated in one of the largest producers and that it is difficult for small producers to stay competitive. Price of farmland in Läänemaa has increased since 2013, both in terms of sales and renting price and for arable as well as grassland. As expected, arable land is more valuable than grassland and this difference tends to be particularly high for renting price in Läänemaa. Land purchases and rental prices are under increasing pressure as vacant land as a key resource for production is running out. When interviewing farmers, their clear concern was the establishment of solar parks in Lääne County as elsewhere in Estonia. It is an attractive and supportive activity, but it plunders a large number of production areas. It is planned to install solar parks on almost 600 hectares in Lääne County. Lääne County is the lowest level in Estonia in terms of the selling price of arable land, the median price per hectare in 2020 was 3 000€ per hectare. According to Statistics Estonia, the rent for arable land in 2018 was 39 €/ha.

In 2020, the farms had the income support payments with 113,17 euros/ha (165,26 euros/ha with payment for agricultural practices beneficial for the climate and the environment), and the premiums for environmentally friendly agricultural production with 83 euros /ha. Rural Development support for management of semi-natural communities is only applicable inside protected areas/Natura 2000 sites. 99% semi-natural habitats are part of protected areas or Natura 2000 sites. Parces where PRIA (Agricultural Registers and Information Board) subsidies had not been applied were considered grasslands out of maintenance. In Lääne County, approximately 34% of grasslands are likely to be out of maintenance (Villoslada et al., 2018). Although there is an obligation to ensure that the ratio of areas of permanent grassland to the total agricultural area declared by the farmers at national level does not decrease by more than a 5% compared to 2015, the risk of a decrease in grassland areas is high. The reason for this is more favourable conditions for intensive agricultural production, for cultivation energy crops or as well for afforestration non-used agriculture land.

### Reference situation for Münsterland

The case study region Münsterland (6752 km²) is located in the north-west of Germany (federal state of North Rhine-Westphalia) and comprises approximately 2.6 million inhabitants and consists of five districts (Steinfurt, Borken, Coesfeld, Recklinghausen, Warendorf) and the city of Münster. The predominantly flat region, which lies between 40 and 200 metres above sea level, has a characteristic landscape. It is a small-structured landscape with open agriculturally used areas and typical landscape elements such as small forests, hedges, wet grassland, small bodies of water, which is referred to as the "Münsterland park landscape". The average annual temperature in the Münsterland is 9 degrees Celsius. The average annual precipitation is about 800 mm, with about 350 mm falling in the main vegetation period (May to September). In the west and east of the region, sandy soils influenced by groundwater predominate, while loamy to clayey soils with better soil quality are found in the centre of the region. The agriculturally shaped landscape is part of a larger intensive livestock farming region where fodder, food and energy crops are grown. The Münsterland energy region is based on extensive renewable energy production through photovoltaic plants, wind power and biogas plants. The region is close to the densely populated Ruhr area, which is an important market for regional products. The demand for regional and ecological products in the city of Münster is based on the social structure (University of Münster, public sector, administration, innovative companies). About 29,000 people are directly employed in agriculture. Agriculture is an important economic factor for the region. The landscape elements in Münsterland thus restrict allocation processes and farm expansions as they are protected by cross-compliance regulations. To minimise the discharge of nitrates, there are further management restrictions in the so-called red zones. In these zones, farmers are required to reduce nitrate losses through adapted fertiliser management and other measures.

In 2020, there were 10,235 farms in Münsterland producing on 385,374 ha of agricultural land. The average farm size was therefore 37.7 ha /LF. The farm types were mainly livestock farms (grazing cattle) with 32 %, finishing farms with 31 %, but also arable farms with 22 %, as well as integrated farms such as crop/livestock associations with 6.4 %, livestock associations with 5.5 %, crop associations with 0.4 % and, to a lesser extent, horticultural farms with 2.1 % and permanent crop farms with 0.2 % of the number of all farms. The number of farms decreased slightly between 2010 and 2020, with the area used for agriculture increasing slightly as well. There were changes between the individual farm types. For example, there were significant annual decreases in the number of farms with livestock, the number of farms decreased between 2010 and 2020 by -5.5 % annually for livestock farms, by -1.3 % for crop production/livestock farms, by -0.1 % for pure livestock farms (grazing cattle) and by -0.03 % for finishing farms. The share of organic farms is low and comprised 1.94 % of farms in 2020. However, this share has increased by 7 % annually between 2010 and 2020. Direct payments and premiums for environmentally friendly agricultural production varied according to farm type and time considered. In 2020, the finishing farms had the highest single farm payments with 300 euros / ha UAA, but the lowest premiums for environmentally friendly agricultural production with 15 euros / ha UAA. The arable farms had the lowest single farm payments of 287 euros / ha UAA, but the highest premiums for environmentally friendly agricultural production of 22 euros / ha UAA. In the sum of direct payments and premiums for environmentally friendly agricultural production, the integrated farms achieved the highest payments in 2020. In 2010, the variation between the farm types was much greater; overall, the livestock farms (cattle) benefited from the highest payments in 2010, due to high single farm payments and high environmental premiums compared to the other farm types.

### Reference situation for Schwarzbubenland

The Schwarzbubenland in the canton of Solothurn, specifically the municipalities of Büren, Gempen, Hochwald, Nuglar-St. Pantaleon and Seewen, serves as research area. The region encompasses an area of 4321 ha, of which 43% are forest, 32% agricultural land (crop and grassland), 17% orchards and landscape features like hedgerows, areas for promotion of biodiversity (BFF), and 6.5% are settlements and infrastructure. The farms manage an average of 24 hectares, mostly in a mixed system of crop production and livestock farming (mainly meat and dairy production). On average, 0.77 GVE* per hectare are kept. The region is traditionally shaped by its standard tree for cherry production. Fruit production is still relevant but decreasing rapidly. Together, the region is populated sparsely and lives off commuters into the agglomeration of Basel.

The area under investigation is shaped by a small-scale mix of forest, agriculture, and settlements, which stretches the Swiss hilly countryside of the Jura mountains. It covers an area of about 43 km^2^ spanning an elevation of 430 to 670 m. The yearly average temperature is 7.7°C and the average precipitation is between 800 to 1000 mm. Yet, the low soil depth and the low water holding capacity of the soil (lime) are clearly hampering the agricultural production.

Generally, the region is populated sparsely. The closely located city Basel, with its economic and structural development, has great influence on the region. Many farms are being led by persons having off-farm jobs (mostly in the city) and doing farm work in the evening or at the weekends. The (few) full-time farmers, on the other hand, can benefit from this proximity to Basel as day trippers show great interest in regional products and direct marketing ex farm can be a remarkable business. Furthermore, the region is of national interest concerning nature and landscape conservation.

### Reference situation for Wienerwald

The area of the Austrian Wienerwald (WW) can be divided into two geological areas. The northern part consists mainly of flysch rock (“Sandstone-Wienerwald”), while the southern part mainly of limestone, which appears near the so-called thermal line (a geological fault line). Average annual temperature amounted to 8.8°C from 1960-2020 and was calculated as 10.4°C for 2021 (Conradt, 2021), which represents a statistically significant temperature trend of +0.53K per decade. Mean annual precipitation increased from 736 mm in 1961-1990 to 773 mm in 1991-2020, but is not statistically significant. Important economic location factors are, among others, the availability of farm labour, input and output prices, subsidies and opportunities for direct marketing. Overall, the development in the Austrian WW region is strongly influenced due to its vicinity to Vienna, Austria’s capital and largest city. Vienna offers many high-income job opportunities, which is a very attractive alternative for many inhabitants of the WW region compared to farming. However, the vicinity to Vienna also offers many direct marketing opportunities and a very large potential customer stock. Due to problems of hay marketing, some farmers founded the “hay stock market”, where hay producers and consumers were connected. Agri-environmental policies play an important role in Austria in general and the in the WW region in particular. The uptake of agri-environmental-climate measures is higher in the WW than the Austrian average (52% of the UAA in WW compared to 41% in Austria, if the participation in the measure "environmentally-sound management (UBB)" in ÖPUL is taken as an indicator). The average agri-environmental premiums per farm (€ 3,453) and per ha (€ 127) are also higher in WW than in Austria (€ 2,619 per farm / € 111 per ha), which suggests a slightly higher uptake of multiple measures in the region. In 2009, the participation in the measure "environmentally sound management" from ÖPUL was 44% of the UAA and the average agri-environmental premiums per farm (€5,507) and per ha UAA (€215/ha) were significantly higher than in 2019. There are no such large differences in the Austrian average (€3,103/farm, €151/ha UAA in 2009). The BR also aims to enhance sustainable regional development, especially related to land use, ecosystem services and biodiversity. Hence, a range of additional awareness-raising and educational programmes are offered by the BR, e.g. the meadows championships. In total, 1237 (1344) main farms existed in the WW in 2019 (2009). The average size of farms increased from 25.6 ha UAA in 2009 to 27.2 ha UAA in 2019. Most farms were fodder crop farms (507), followed by ruminant farms (309). Although most of the WW is forest (70%), permanent grassland (12%) play an important role in the area, e.g. for food and fodder production, leisure and recreation, and biodiversity. The grassland and meadows, as well as the forests, are frequently visited by the Viennese for recreation also leading to several conflicts among different users (e.g. farmers, dog owners, mountain bikers). The following table X gives an overview on regional land use and its development since 2015. Overall, permanent grassland declined by 13.5% and cropland increased by 16.7%. General trends are extensification of grassland management, intensification and abandonment of land. Environmental/ecological interviewees described that the most valuable grasslands for biodiversity have been and are being abandoned - indicating the need for more targeted policies.

## B.3 Narratives

In the following, first summaries of the three case study specific narratives are presented for each of the three XX-Agri-SSPs, followed by the narratives of each case study.

### XX-Agri-SSP1: Regional agriculture on sustainable paths

*Population and Urbanization*

European citizens have a high social and environmental awareness and share a strong demand and commitment to sustainable agricultural land use. Overall, it is more attractive to live in smaller towns in rural areas in some case study regions. However, competition for land increases in the vicinity of larger cities such as Münster, Basel, or Vienna because urban areas still attract people. This manifests in high land prices, also driven by the high purchasing power of the urban population. Farming is more produce agricultural goods while maintaining high ecological sustainability. The farmers, thus, share high commitment and gain fulfillment by providing multifunctional landscapes.

*Economy*

There is a high demand for regionally grown food that allows farmers to profitably sell e.g. orchard cherries, a regionally distilled liquor made of cherries in Schwarzbubenland, or meat, fruits, and vegetables in the Wienerwald. Farmers mainly sell their goods on regional markets or farm shops, especially because consumers ask for closer consumer-producer relationships and show a high interest in sustainable production processes that deliver multiple ecosystem services. In general, the demand for meat decreases, and products respecting animal welfare are consumed by the case study inhabitants. This leads to price increases and thus the competitiveness of grazing cattle and sheep (e.g. in Wienerwald or Lääne county). There is a great interest in reintegrating food production into everyday life which allows people to foster their knowledge and relationship with nature. This can be seen, for example, in active help with vegetable production and harvesting (i.e. community supported agriculture). Labor productivity improves through better training and investment in technology. The productivity of agricultural land is affected on the one hand by the decrease in the use of inputs and on the other hand by technological development.

*Policies*

There are strict international and national regulations for sustainable, biodiversity-friendly, and resource-efficient land use. To preserve biodiversity and ecosystem services, the government supports high investments in agri-environmental-climate measures. The policy focus is on multifunctional land use, considering historical biodiversity-friendly forms of management and implementing result-based measures. Accordingly, there are high subsidies for extensive agricultural management, ecological infrastructure (like trees, hedges) as well as measures to improve the connectivity of wildlife habitats. At the same time, policies aim to ensure strong nature protection in areas that require no disturbances to safeguard biodiversity.

*Technology*

Environmentally friendly agricultural technologies are being developed taking into account the entire production cycle. The development and use of technologies and renewable energy sources that support organic farming are important. Farmers and food processors are aware of the environmental impacts of their production activities (greenhouse gas emissions or biodiversity impacts) and are able to measure and, in case of positive externalities, market them. The introduction of new technologies is enhanced by good cooperation between the public and private sectors. Learning from the best available practices is promoted and supported, i.e. innovative farms cooperate well with each other. New biological and environmentally friendly management practices are developed and are efficient in controlling pests and diseases (e.g. the spotted-wing drosophila in SB) without high costs for farmers.

*Natural Resources and Environment*

The inhabitants of Vienna and the WW, Münster and the ML as well as of Basel and the SB continue to use the forests and cultural agricultural landscapes as a recreational space. However, the increasing environmental awareness of the population contributes to fewer conflicts than before. For example, there are dedicated areas for recreational use. Energy production, e.g. through ground-mounted photovoltaic plants or wind turbines, is strongly restricted in open spaces to avoid potential negative consequences for biodiversity and competition for land in some case studies, including the Wienerwald. However, agri-PV, i.e. photovoltaic systems which still allow agricultural production on their installation sites, are fostered. In ML, existing biogas plants play a reduced role as a flexible producer and storage of energy in times of low production from solar and wind energy sources.

### XX-Agri-SSP2: Regional agriculture on established paths

*Population and Urbanization*

Population continues to grow in particular in urban agglomerations in the case studies. The regional policies follow the principle of “managed shrinkage”, especially in Estonia. Regional governments maintain only the necessary infrastructure in smaller settlements and rural areas; the infrastructure is reduced as settlements become depopulated. There is a growing awareness of environmental problems and a growing interest in social and environmental responsibility in the agricultural sector. The participation of the younger generation in agriculture is encouraged and the share of people with agricultural education grows.

*Economy*

Price levels and inputs to agricultural production are stable, mainly due to technological developments, open markets, and a lack of abrupt changes between the agricultural industry and producers. The prices of natural resources, such as land and water, rise as demand for them increases and the quality and quantity of resources decrease. The agricultural workforce (both skilled and unskilled) is stable due to international immigration and improved working conditions, e.g. in Lääne county or the Münsterland region. Labor productivity rises gradually as technologies evolve and education levels rise in all case studies.

*Policies*

The share of direct agricultural payments in the EU's common agricultural policy (i.e. income support) as well as the Swiss agricultural policy is declining, but area-based payments are still present. The importance of environmental subsidies and investments increases. Mechanisms to support rural development remain at current levels, but they are not sufficient to reduce rural depopulation. Rather, this is promoted by investments in technology support for non-agricultural activities and jobs. Investments in digitization and precision farming are supported.

*Technology*

The development of existing innovative technologies (e.g. precision farming) and new technologies that support resource-efficient management (e.g. optimal use of fertilizers, pesticides, and feed) continues. The uptake of new technologies also increases, partly due to better education and skills of farmers.

*Natural Resources and Environment*

The pressure on land in the case study regions intensifies. For instance, there will be more competition for agricultural land, such as land demand for the construction of new solar or wind parks in LE or WW. At the same time, recreational use of green spaces within cities or of cultural landscapes and forests near urban areas, such as the SB and WW region, continues to increase. This leads to conflicts between different users of meadows, forests, or other green spaces. In ML, existing biogas plants play a constant role, also as a flexible producer and storage of energy in times of low production from solar and wind energy sources.

### XX-Agri-SSP5: Regional agriculture on fossil-fueled, high-tech paths

*Population and Urbanization*

The population grows in all case studies but exclusively in urban agglomerations. Society and farmers share a very high affinity for technology and accept deteriorations of the environment to stimulate economic growth. Citizens cannot relate to nature anymore and share the perspective that most ecosystem services can be substituted with technological solutions and innovations.

*Economy*

Agriculture is moving towards industrialization and global market integration. With cheaper inputs and technological innovation, higher productivity is achieved. The share of skilled labor in agriculture is increasing, contributing to high labor productivity. Open markets and thus higher competition, as well as low environmental standards contribute to low market prices for food. As a result, agricultural land is segregated between the intensification of (very) profitable agricultural areas and the abandonment of non-profitable areas. For example, the steep slopes in SB or WW, nowadays managed as extensive meadows and orchards, are no longer perceived as valuable by society and thus lose subsidies and finally attractiveness for active management. Family farms are less competitive because higher incomes are achieved in other sectors and the labor demand in nearby cities is very high. However, new types of farms and farming are being established, e.g. larger corporate farms and indoor farming of edible insects as a new type of food or animal feed.

*Policies*

In most case studies, subsidies, whether income support through direct payments or agri-environmental-climate-measures, are abandoned. The remaining subsidies are channeled into financial instruments (such as low-interest long-term loans) to ensure liquidity. Some public investments are maintained to promote agricultural education to boost labor productivity. Most regional land use restrictions, such as the Flora-Fauna-Habitat directive, will be relaxed as nature per se is no longer valued by society and settlements will be allowed to grow in all case studies.

*Technology*

Society and farmers strongly favor the development and use of new technologies. Technology-aspects are valued in the education and advanced training of farmers. Thus, new technologies conquer the case studies to achieve high profitability and reduce labor inputs. Larger fields managed by e.g. precision farming and controlled traffic farming dominate the remaining agriculturally managed landscapes.

*Natural Resources and Environment*

The reduction of most environmental standards facilitates the use of natural resources, such as land and water. Technological solutions are fostered to improve the water supply in the SB and the WW, e.g. by constructing small water tanks and artificial reservoirs. Smart land amelioration systems are applied along with other measures to increase field sizes. Some rural areas are still frequently visited by urban residents, e.g. of Münster city, but exclusively for recreational purposes. Thus, the respective leisure opportunities, e.g. amusement parks, are being greatly expanded but somewhat separated from the industrial farming of the land. In ML, biogas plants are slightly increasing due to higher livestock numbers and the corresponding increase in slurry.

### Läänemaa

#### LE-Agri-SSP1

The concentration of people in larger cities continues, but it becomes attractive to live outside the core city, which gives people a better access to social and technical infrastructure. Values are shifting towards greater social and environmental awareness. Agriculture is becoming an attractive sector to work for young and educated people, and the age structure in the agricultural sector is relatively balanced. Farms are of different sizes and orientations: the keyword is cooperative activity. Natural resources are valued, which leads to an increase in the price of natural resources. The agricultural workforce is stable due to smaller farms, lower production intensity and every effort is made to keep the production supply chain as short and transparent as possible ("farm to fork production" model). The meat of beef cattle raised in the semi-natural communities of Lääne County is preferred by the consumers because it supports biodiversity-friendly agriculture. Farmers and consumers have more direct contacts. Labor productivity should improve through better training and investment in technology. The productivity of agricultural land is affected on the one hand by the decrease in the use of inputs and on the other hand by the development of technology. A certain group of consumers want to consume more environmentally friendly products and are willing to pay more for it. Demand for animal products is declining as consumers are affected by attitudes towards consuming less animal protein. More money from direct payments is given to address environmental issues. Support will be decoupled from production and will be directed to a greater extent to greening measures and environmental policy. The state buys environmental services from producers. Rural development support is not only an investment to increase the sustainability and competitiveness of rural sectors, but should also enable the provision of public services and goods to society. The investments will support environmentally friendly practices and technologies and will lead to more environmentally friendly production. Environmentally friendly agricultural technologies are being developed that take into account the entire production cycle. The development and use of technologies and renewable energy sources that support organic farming is important. The introduction of new technologies is enhanced by good cooperation between the public and private sectors. We learn from the best available practices, i.e. innovative farms are cooperating well among each other. The condition of ecosystem services in agricultural landscapes is improving: on one hand, via more environmentally-friendly subsidies but on the other hand, also producers themselves value other ecosystem services in addition to production. Land use as a whole is more environmentally friendly and traditional landscape structure has been preserved at least in part, biodiversity in the agricultural landscape is maintained or increased. The challenges posed by climate change in agriculture and food systems are diminishing. There are more visionary farmers who are willing to do and are actually doing more than the minimum regulations require. Manufacturers are aware of the footprint of their production activities (carbon footprint) and are able to measure and market it.

#### LE-Agri-SSP2

In 2020, there are 20,444 inhabitants in Lääne County. The number of permanent residents of Lääne County will decrease further, people and resources will continue to be concentrated in and around larger cities, such as the Tallinn city region, and to a lesser extent also in the vicinity of Tartu and Pärnu. The state's regional policy follows the principle of “managed shrinkage”. Regional governments maintain only the necessary infrastructure in smaller settlements and rural areas; the infrastructure is reduced as settlements become depopulated. The population of Lääne County is aging and decreasing. The projected population in Lääne County in 2045 is projected to be 14,896. There is a growing awareness of environmental problems and a growing interest in social and environmental responsibility in the agricultural sector. The participation of the younger generation in agriculture is encouraged and the share of people with agricultural education in the sector is growing. Farms are becoming larger, more profit-oriented, which is a prerequisite for their competitiveness in an integrated market. The small producer dies out. Price levels and inputs to agricultural production are stable, mainly due to technological developments, open markets and a lack of abrupt changes between the agricultural industry and producers. The prices of natural resources, such as land and water, are rising as demand for them increases and the quality and quantity of resources decreases. The agricultural workforce (both skilled and unskilled) is stable due to international immigration and improved working conditions. Labor productivity is gradually rising as technologies evolve and education levels rise. The productivity of agricultural land remains the same. Price levels and product diversity are stable. The availability of high-quality raw materials to people is low, high-quality products are exported from the region, including Estonia. The share of direct agricultural payments in the EU's common agricultural policy is declining, but area-based payments are still part of the policy. The importance of environmental subsidies and investments is increasing. Mechanisms to support rural development are at current levels, but they are not sufficient to reduce rural depopulation. Rather, investments in technology support non-agricultural activities and jobs. Investments in digitization and precision farming will be supported. The development of existing innovative technologies (e.g. precision farming) and new technologies that support resource-efficient management (e.g. optimal use of fertilizers, pesticides and feed) will continue. The uptake of new technologies is also increasing, partly due to better education and skills of farmers. The pressure on land use in Lääne County is intensifying. There will be more competitive developments for agricultural land use, such as the construction of new solar parks. Biodiversity in agricultural landscapes is declining, the condition of ecosystem services is deteriorating and the landscape structure is being simplified. In agricultural production, there are more problems with invasive species and plant diseases. The situation of coastal waters is deteriorating, fish stocks are declining. The living environment and, as a result, human health are deteriorating.

#### LE-Agri-SSP5

The importance of environmental concerns is recognized foremost because it is important for maintaining human well-being and consumption levels. Although the population will continue to be concentrated in cities, it will take place within the labor market areas of larger county towns. Lääne County will become a specialized and attractive region with places to live and work. Agriculture is moving towards industrialization and global integration. With lower inputs and smarter decisions, higher productivity is achieved. There is an intensification, but it is a sustainable process that saves the environment. By integrating production, pre-processing and processing, producers are able to make most out of raw materials in the value chain. Input prices are falling due to the rapid development of technology. Fossil resources become cheaper as alternatives to them emerge on the market. The share of skilled labor in agriculture is increasing (need for skilled workers and analysts). Productivity of agricultural land is increasing and labor productivity is high. The price level is stable, but the key word is risk management. Sales are made through online platforms. Short supply chains are not yet conceivable for large-scale agriculture. The consumer wants to know where the raw material comes from and what the environmental footprint of the product is. It is possible to calculate the carbon footprint of the product and environmental impacts per product unit. There are less direct area- and production-based subsidies: the majority of policies are moving towards development subsidies. Subsidies are channeled into financial instruments (such as low-interest long-term loans) to give producers access to the financial investment needed in the sector. Technological developments are moving in the direction that the provision of public services to society will not be reduced or negatively affected. The state buys ecosystem services from the producer, such as CO2 quota compensation for the producer, provided that the producer is able to sequester carbon in the soil. Public investments are declining and are mainly focused on promoting agricultural education. The development of education and knowledge and knowledge transfer are important. Very strong focus on the development of new technologies, especially in terms of the efficiency and added value of the production process (e.g. “smart barn”, smart land amelioration, smart irrigation systems). Society and farmers strongly favor the development and use of new. Technology-aspects are valued in the education, training and further training of farmers. Precision farming, smart farms, etc. help reduce regional pressures on the agricultural landscape and greenhouse gas emissions, and help adapt to climate change. Land use intensity is increasing, but diffuse pollution emissions are expected to decrease. If technologies are not supported alongside green infrastructure, biodiversity will be reduced and the landscape structure will not change or become even more homogenic.

### Münsterland

ML-Agri-SSP1

The narrative is based on the "Sustainable Agricultural Pathway in 2050 [Eur-Agri-SSP1] " and is implemented in Münsterland as "Diversified Sustainable Agriculture with Social Consensus".

Agriculture in Münsterland is becoming more environmentally oriented as the social and environmental awareness of citizens reaches a high level. This also leads to a positive image of agriculture. Land and labour productivity is increasing due to the educational level of farmers and technological progress. Farm structures are diversified (small and large producers, cooperatives, innovative start-ups), the average farm size is developing moderately. The demand for meat is decreasing, while the demand for high-quality and regional plant products is increasing.

Measures for environmental, climate and animal protection are based on cooperation between the public and private sectors and aim to increase resource efficiency, protect nature, save energy and reduce environmentally harmful impacts. Direct payments under the first pillar of the CAP are being abolished altogether in favour of large payments under the second pillar. Prices for agricultural products increase and are also accepted due to ecosystem services. In addition, the private sector financially supports measures to improve biodiversity and environmental services.

Environmentally oriented technologies lead to lower consumption of natural resources. Technologies and the high level of education of land users lead to high labour and land productivity. Due to the increasing share of organic farming and improvements in conventional agriculture, the use of chemical pesticides and mineral fertilisers decreases and is supported by innovative technologies whose use is promoted by public infrastructure investments. Substrate for the remaining biogas plants is produced more environmentally friendly.

The lower demand for meat products as well as the diversification towards the production of regional, high-quality products (including organic farming) have positive external environmental effects (e.g. more legumes). Nitrate discharge decreases, while the share of grassland can be maintained due to land-based livestock production. The share of green infrastructure (e.g. hedgerows, small water bodies, individual trees, groups of trees and near-natural strips in fields) can be increased due to rising financial resources and investments.

ML-Agri-SSP2

The narrative is based on the "Established Agricultural Pathway in 2050 [Eur-Agri-SSP2]" and is implemented in Münsterland as "larger scale agriculture with the same policies". This path describes the continuation of the current situation in Münsterland. Due to the slow but steady change in society's environmental awareness, agriculture is faced with societal demands such as taking more environmental and social responsibility. Increasingly, however, it is unable to meet these demands, as the societal demand is not linked to any significant change in European agricultural and environmental policy. In the Münsterland region, this fundamental conflict occurs above all in animal husbandry and conventional agriculture. Society's view of agriculture remains critical and cannot be improved because of the basic conflict described. The interaction between rural and urban areas remains stable. Farm succession is threatened. The agricultural sector faces stagnating to slightly increasing land productivity and slightly increasing labour productivity, with stable price levels for products and inputs, but slight shifts in food preferences towards regional food and protein alternatives. However, demand for meat is not declining significantly. Farm sizes in livestock production (especially pig farms) continue to increase at the pace of structural change to date. Political changes are not really taking place, i.e. the EU's Common Agricultural Policy (CAP) retains the two-pillar structure, the diverse support schemes address different and partly contradictory objectives of increasing efficiency and protecting environmental and social standards. Technological development is moderate but concentrated on resource-efficient technologies. Environmental standards are improving moderately but are limited in scope. Due to the persistence of intensive forms of land use and little effective agri-environmental and climate protection measures, negative environmental effects of agriculture persist: increasing demand and competition for agricultural resources, decreasing diversity of landscapes, decreasing quality of green infrastructure, decreasing biodiversity, and continued nitrate discharges. Conflicts of use occur between different land users (transport and settlement vs. agricultural land), but also between different agricultural uses (bioenergy vs. food, arable land vs. grassland). The loss of agricultural land is unabated and land prices are rising.

ML-Agri-SSP5

The narrative is based on the "Market Liberalisation Pathway in Agriculture in 2050 [Eur-Agri-SSP5]" and is implemented in Münsterland as "Globally liberalised agriculture based on finite resources and fossil energy". Increasing reliance on market liberalisation with strong links to the global market characterises this scenario, with great emphasis on technological progress. Finite resources continue to be heavily exploited and fossil energy continues to be used on a large scale. The perspective of agriculture is changing rapidly due to accelerated structural change with increasing specialisation and orientation towards global markets. Society's view of agriculture is increasingly characterised by a low level of understanding. Land productivity is rising due to accelerated diffusion and increasing acceptance of technological innovations, which is also changing the job profiles of farmers (even higher training standards). Farm labour costs are rising while productivity is high, due to a high degree of automation with robotics. Relative prices for agricultural inputs are falling due to technological progress with high use of fossil resources. Münsterland is undergoing an accelerated structural change with increasing specialisation and orientation towards global markets. While public agricultural investments are drastically reduced, the role of the private sector is increasing, e.g. in the form of start-up companies. Demand structures are changing towards diverse diets at high health quality levels. The drastically reduced EU payments (direct payments, 2nd pillar) with declining EU environmental standards are only compensated to a small extent by the promotion of individual ecosystem services, such as the preservation of biodiversity. Agricultural production is characterised by a rapid diffusion of technological progress, e.g. advances in breeding, agricultural construction and machinery, and integrated farm management systems. Effective logistics (e.g. traceability systems) and marketing technologies are increasingly used. However, it may also mean that currently controversial technologies (e.g. genome editing, Crispr-CAS) are applied more widely. Environmental improvements could potentially be achieved with rising food quality standards and high technological levels. However, this potential cannot be exploited and can only be used as a side effect, as the focus of technology development is aimed at an economic optimum. This can result in unintended negative externalities that are not contained by appropriate environmental regulations. However, it can also result in positive externalities that contribute to the improvement of some environmental aspects, e.g. through high quality standards of food and efficiency improvements in the use of resources that were primarily introduced for marketing reasons and to reduce costs.

### Schwarzbubenland

#### SB-Agri-SSP1

In a CH-Agri-SSP1 scenario, urbanization continues while the overall population size stagnates. Environmental awareness and sustainability are becoming more important, and it is becoming attractive to live in smaller towns in rural areas. These areas provide a high-quality infrastructure and good job opportunities which allow a high quality of life. Farming is becoming more popular and there are now many young and innovative farmers to whom it is important to efficiently produce agricultural goods while maintaining high sustainability. There is a high demand for regionally grown food that allows farmers to profitably sell orchard cherries as well as regionally distilled liquor made of lesser quality cherries. Farmers mainly sell their goods at regional markets or farm shops, which makes them lose less money to big suppliers. There are strict international and national regulations for sustainable, biodiversity-friendly and resource efficient land use. To preserve nature's contributions to people, the government supports high investments in environmentally friendly agricultural management. Accordingly, there are high subsidies for extensive agricultural management, ecological infrastructure (like trees) as well as measures to improve connectivity of wildlife habitats. These policies lead to an extensification of the study region. This can be seen with smaller field sizes, more orchard trees and more flowering meadows with low mowing and irrigation pressure. In addition, there are now new sustainable technologies with no or lower inverse effects on wildlife, such as, for example, controlled traffic farming. New biological and environmentally friendly substances have been developed and are efficient in controlling the spotted-wing drosophila without high costs for farmers. This leads to decreased yield losses, especially for orchards. In this scenario, environmental awareness of the population has grown. Power generation has become more resource efficient, with a focus on sustainable and renewable energies such as solar, wind and water energy.

#### SB-Agri-SSP2

In a CH-Agri-SSP2 scenario population and settlements will slightly grow. Legal restrictions to protect landscape scenery are and will be in place. They will preserve the regional character of small villages in mosaic to rural landscapes. The number of inhabitants will stay the same or slightly increase as the spatial proximity to Basel allows for commuting between work and home. Economy in CH-Agri-SSP2 will be business as usual. That means, several supporting schemes are in place to promote farming activities, biodiversity conservation and diversification. Hence, there will be land use conflicts between urbanization (more infrastructure / tourism) and agriculture as well as between agriculture and biodiversity promotion and nature conservation. The number of part-time farmers will increase, while the number of full-time farmers will slightly decrease. Direct selling and farmers markets might show a huge potential for the regional economy and the production of niche products. In Schwarzbubenland, land use policy is very restricted due to national conservation standards (“Landscapes and Natural Monuments of National Importance”). These regulations will remain in place. New technologies will slowly arrive and be applied in the region (moderate technological advances). Application of robotics and automatisation of grassland management might be realistic within the next few years. The high number of part-time farmers limits the financial benefits of technology application and therefore a switch to a more technological driven agriculture is not realistic. Environmental supporting schemes are in place and will be further supported. Especially the preservation of orchards trees, as a landscape character of the region, will be put in the spotlight. In addition, hedges and biodiversity in arable land will be supported.

#### SB-Agri-SSP5

In a CH-Agri-SSP5 scenario population and settlements will grow. Legal restrictions reducing building activities to protect landscape scenery - as they are currently in place - will be removed. In addition, the spatial proximity to Basel (capital city) will increase the commuter volume and therefore the total number of inhabitants will rise in the region. Environmental standards will be reduced in all fields of management. In agriculture, this will be followed by a segregation between intensification of (high) profitable agricultural businesses and abandonment of non-profitable areas. For example, the steep slopes in Schwarzbubenland, nowadays managed by extensive meadows and orchards, will not be profitable anymore and result in an increase of forest area. On the other hand, the flat plateau region will be favoured for arable production. While the thin and permeable soil does not allow for high production levels nowadays. Soil improvement by soil refill will take place and will raise the production level. This will go hand in hand with the need and the installation of irrigation water systems. Small water tanks and artificial reservoirs will be constructed in the region. In liberalized markets, the total number of full-time farms will decrease, while the part-time farmers will give up. In Schwarzbubenland, land use policy is very restricted due to national conservation standards. The region is part of the national inventory of “Landscapes and Natural Monuments of National Importance”. Hence, in a CH-Agri-SSP5 scenario these restrictions will be relaxed, and settlements will be allowed to grow. New technologies will conquer the region. In line with more full-time farmers, the leading maxima “achieving high profitability and reducing labour inputs” will result in higher technology application per farm. Larger fields managed by precision farming and controlled traffic farming will be present in the future. Grassland management will be done by robotics. Environmental standards will be reduced in all fields of management. This will result in an abandonment of biodiversity promotion and biodiversity promotion areas due to high costs and reduced agricultural productivity. Extensive meadows and traditional fruit orchards will be eliminated in favour of forest or intensive grassland management. Hedges and old single trees will be cleared to facilitate technology use.

### Wienerwald

Narratives for entire Austria have been separately developed and informed the narratives for the Wienerwald (see Karner et al. 2024). Below, only the summaries developed within SALBES for the Wienerwald region are presented.

#### WW-Agri-SSP1

The popularity of the WW as a place to live continues to rise. This leads to a stronger increase in land prices than in the rest of Austria, also driven by the fact that the purchasing power in Vienna and the WWis higher than in other areas of Austria. However, in order to keep new construction low, there are strict regulations regarding land consumption and housing densification. In addition, energy production, e.g. through photovoltaics or wind turbines, is strongly restricted on open spaces, also to minimise competition for land. The regional administration of the WW implements strongly result-oriented measures, in addition to the Austria-wide measures regulated by ÖPUL. The focus is on multifunctional land use, taking into account historical biodiversity-promoting forms of management. This means that the aim is to enable food production, the provision of a variety of ecosystem services and the preservation and support of biodiversity. Grazing communities and other management communities are promoted to keep extensive grassland management attractive. In addition, there are also "Green Care" offers for meadow management. Furthermore, there are subsidies for grazing, also by different animal species such as alpakas, llamas or rare beef species. The demand for meat is decreasing in Austria and only animal welfare products are consumed by Austria’s inhabitants. This increases the prices and thus the competitiveness of grazing cattle and sheep from the WW, especially since the Viennese population has a strong demand for regional products. This is also reflected in a strong increase in direct marketing, the implementation of which is also supported by municipalities. However, the Viennese population does not only demand sustainable meat from extensive meadow farming in the WW, but also fruit and vegetables, for example. There is a great interest in bringing the relationship to food production back to the centre, which can be seen, for example, in active help with vegetable production and harvesting (i.e. community supported agriculture). The inhabitants of Vienna and the WW continue to use the area as a recreational space. However, the increasing environmental awareness of the population contributes to fewer conflicts than before. For example, there are separate recreational use areas. Horse riding maintains its attractiveness here, which leads to higher rental prices.

#### WW-Agri-SSP2

The WW are exposed to strong land pressure. On the one hand, urbanisation is increasing, leading to an expansion of the city of Vienna into the WW. On the other hand, the WW remains a popular (second) residential area due to its proximity to the city. In addition, energy production is increasing, also on open spaces, e.g. through agro-photovoltaics and the expansion of wind parks. The Viennese still like to relax in the WW and on the meadows of the WW. This leads to ongoing conflicts with the use of agricultural meadows. Dog owners, walkers and cyclists show little consideration for agriculture and private land. They take the view that their recreational use is more important than agricultural use. All in all, the attractiveness of meadow use, especially pasture farming, is therefore declining. In turn, the opportunities for agriculture increase, especially as access to resources, e.g. to water for irrigation in the north of the WW, becomes easier.

#### WW-Agri-SSP5

The WW is frequently used by the residents of Vienna, especially for recreational purposes. For this purpose, the offer is being greatly expanded. For example, there is a newly built golf course, archery courses, new mountain bike trails, riding arenas and trails or climbing gardens. Small-scale land use, especially the use of meadows, is being completely abandoned, mainly due to the lack of subsidies. The only regional consumers of hay are equestrian farms, which have high quality requirements. In general, only a few large farms remain, which cultivate large fields with autonomous tractors, etc. The use of pasture is hardly interesting in the WW, due to the heavy recreational use. Family farms are less attractive because higher incomes are achieved in other sectors and the labour supply in Vienna is very high. However, new types of farms are being established, e.g. for insect production for new types of food or as animal feed.

## B.4 LUMPs

### Läänemaa

Table SM.B 4.1: LUMPs as specified for Läänemaa, Estonia for all three SSPs

| LUMP classifi-cation | LUMP description | | SSP1 | SSP2 | SSP5 |
| --- | --- | --- | --- | --- | --- |
| New crops, livestock, and land-uses | Grain maize | | x | x | x |
|  | Chickpeas, lentils (have been used also historically in Estonia) | | x | x | x |
|  | Winter barley | | x | x | x |
|  | Some fodder hay plants (re: animal husbandry) | | x | x | x |
|  | Soy | | x | x | x |
|  | Sweet Potato | | x | x | x |
| New technolo-gies | Biomethane production and centers at cattle and beef farms + Tractors using biomethane; more use of other alternative fuels | | x |  |  |
|  | Robots for fertilization/plant protection | | x |  | x |
|  | Smart barns aimed at energy efficiency, GHG reduction, optimal air circulation, heat recovery ventilation, etc. | | x |  | x |
|  | Local/regional photovoltaic/wind energy production + energy cooperatives, loading stations for robots | | x |  |  |
|  | More extensive use of minimized tillage, direct sowing, reducing of GHG emissions also via animal husbandry practices, e.g. feeding ratios | | x |  |  |
|  | Precision farming: precise fertilizer spreaders/plant protection spraying mechanisms; N-sensors; infrastructure (weather stations) | |  | x |  |
|  | Controlled traffic farming | |  | x |  |
|  | Use of biocompost; Slurry separation (learning, developing methods) | |  | x |  |
|  | Smart land amelioration systems: regulated (remote-control) | |  |  | x |
| New biodiversity practices | antaln green areas as a part of ecological networks | va uable | x |  |  |
|  | grasslands, connected with smaller grasslands and green corridors, bordered with streams, wetlands, hedges.) | |  |  |  |
|  | Leaving wider uncultivated grass margins to benefit birds and pollinators around arable land | | x | x |  |
|  | Creation of feeding fields for migrating birds | | x | (x) |  |
|  | Paludification with increasing water table level | | x |  |  |

### Münsterland

Table SM.B 4.2: LUMPs as specified for Münsterland in Germany for all three SSPs

| LUMP classifi-cation | LUMP description | SSP1 | SSP2 | SSP5 |
| --- | --- | --- | --- | --- |
| New crops, livestock, and land-uses | Durum wheat | x | x | x |
|  | Sunflower | xs | x | x |
|  | Soybean and other legumes (field bean, pea, lupine, alfalfa, chickpea, vetches) | (higher shares due to reduced livestock numbers; lack of N | x | x |
|  | Fruits & vegetables for regional markets   - Vegetables: main representatives are carrots, spinach, onions/leeks - Commercial crops: strawberries - Permanent crops: asparagus; fruit | higher share | lower share |  |
| New techno-logies | Photovoltaic systems and biogas plants, types |  |  |  |
|  | 1. **open field PV** |  | x | x |
|  | 2. **biodiv-solar parks 1,2** | higher share | lower share |  |
|  | 3. agri-PV 1, 2, unterschiedliche Anteile | SSP1 | SSP2 |  |
|  | - **Agri-PV in strips with flowering strips** 1,2 mehr | lower share | higher share |  |
|  | *- Agri-PV with grazing (vertical, slightly sloped, use: grazing cattle, sheep)* | *discussed but describes a specific, not standard situation* | |  |
|  | *- Agri-PV with cultivation of special crops (e.g. blueberries)* | *discussed but describes a specific, not standard situation* | |  |
|  | 4. **biogas plants expansion** with regard to storage and flexibilization of power output | reduced use (lower share) | constant use (constant share) | slight increase, corresponds with slurry amount |
|  | Slurry application (N, P issues) |  |  |  |
|  | 1. feed composition according to growth stage (set as standard, not to be modeled, nitrate content if necessary) | x | x | x |
|  | 2. use slurry as substrate for biogas plants (set as standard) | x | x | x |
|  | 3. **injection of slurry** instead of drag hose or drag shoe application | x | x | x |
|  | 4. use of **nitrification inhibitors** |  | x | x |
| New bio-diversity measures | Measures on arable land |  |  |  |
|  | 1. annual and perennial flowering mixtures, each with and without biogas use. | x | x |  |
|  | 2. extensive cereal cultivation | x | x |  |
|  | 3. pole bean-maize mixture | x | x |  |
|  | 4. maize cultivation with clover/grass undersowing | x | x |  |
|  | 5. rapeseed with double row spacing (allowing mechanical weeding instead of pesticides) | x | x |  |
|  | Measures on grassland |  |  |  |
|  | 1 Extension of horse keeping (more pastures, late mowing on meadows) 1 | x |  |  |
|  | 2 Extension of sucker cow systems 2 |  | x |  |
|  | 3 Strips of older grass patches 2 |  | x |  |
|  | Measures for supporting “Green Infrastructure” |  |  |  |
|  | Establishment of a network to support/maintain green infrastructure | x | x |  |
|  | Certain share of landscape elements per farm | 10%, | 5% | 1% |

### Schwarzbubenland

Table SM.B 4.3: LUMPs as specified for Schwarzbubenland, Switzerland for all three SSPs

| LUMP classification | LUMP description | SSP1 | SSP2 | SSP5 |
| --- | --- | --- | --- | --- |
| New crops, livestock, and land-uses | Soy, maize, sunflower | x | x | x |
|  | New crops/breeds varieties | x | x | x |
|  | New hedges/tress |  | x |  |
|  | Intercropping, agroforestry |  |  | x |
| New technologies | Tractors using bio-fuels or other alternative fuels | x | x |  |
|  | Light-weight, small e-tractors, robots |  | x | x |
|  | Standard Precision Farming (focus on Fertilization/Pest Control to maximize yields) |  |  | x |
|  | Biodiversity-friendly precision farming (focusing on spatially and temporally targeted management to minimize biodiversity impacts, e.g, optimized mowing dates, spatially differentiated inputs | x |  |  |
|  | Use of biocompost, slurry separation (learning, developing methods) |  | x |  |
|  | Controlled traffic farming | x |  |  |
|  | Use of drones to protect game etc | x | x |  |
|  | Irrigation |  | (x) | x |
|  | Land consolidation |  |  | x |
| New biodiversity measures | Certain share of landscape elements per farm | 3% greening in arable fields, 7% in total | 3% greening in arable fields, 7% in total |  |
|  | Rewilding of abandoned farms (segregation of intensified and abandoned farms) |  |  | x |
|  | Establishment of a network to support/maintain green infrastructure | x | x |  |

### Wienerwald

Table SM.B 4.4: LUMPs as specified for the Wienerwald, Austria for all three SSPs

| LUMP classification | LUMP description | SSP1 | SSP2 | SSP5 |
| --- | --- | --- | --- | --- |
| New crops, livestock, and land-uses | Durum wheat | x | x | x |
|  | Soy | x | x | x |
|  | Conversion of arable land in grassland | x | x |  |
|  | New crops/breeds (e.g. stress/drought-resistent varieties of pumpkins and potatos) | x | x | x |
|  | Diversification of animals (e.g. ostrich, angus cattle) | x | x |  |
|  | Land consolidation |  | x | x |
|  | Open land preservation for recreational uses (e.g. golf course) | x | x | x |
| New techn-ologies | Light-weight, small e-tractors, robots | x |  |  |
|  | Tractors using bio-fuels or alternative fuels |  | x |  |
|  | Standard Precision Farming (focus on manure management, fertilization and pest control to maximize yields and minimize losses) | x | x | x |
|  | Biodiversity-friendly precision farming (focusing on spatially and temporally targeted management to minimize biodiversity impacts, e.g, optimized mowing dates, spatially differentiated inputs | x | x | x |
|  | Agri-photovoltaic plants (bifacial) | x |  |  |
|  | Open field Photovoltaic plants and windparks |  | x |  |
|  | Use of drones to protect game etc | x | x |  |
|  | Glass houses |  | x | x |
|  | Irrigation |  | x | x |
|  | Track surveying and alignment of fields |  |  | x |
| New biodiv-ersity measures | Rewilding of abandoned farms (segregation of intensified and abandoned farms) | x | x | x |
|  | Establishment of a network to support/maintain green infrastructure | x | x |  |
|  | Certain share of landscape elements per farm | 10% | 5% | 1% |

## B.5 LBAs

First the full joint list of LBAs depicted in the following table B5.1. B5.2-B5.7 show case-study specific tables of LBAs, which was developed for the German case study Münsterland. Regional priority was scored independently by each case study team on a scale from 1 (low) to 3 (high). These scores were first averaged across the four regions to obtain an average regional priority index. The applicability index and the impact on biodiversity index were built using assessments by the entire research team. The final evaluation score was then calculated as the arithmetic mean of three components: (i) the average regional priority index, (ii) the applicability index (1–3), and (iii) the biodiversity impact index (1–3).

Table SM.B 5.1: Overview of all specified LBAs in all four case studies (ND = not discussed)

| **LBA (Land-use biodiversity action) description** | **Applic-ability: 1 - not possible, 2 - difficult, but possible, 3 - easily applicable** | **Impact on biodiv-ersity: 1 -** **little, 2 -** **medium, 3 - substantial** | **Prioriy for** **WW (AT): 1 -** **little, 2 -** **important, 3 - very important** | **Priority for LE (EE): 1 -** **little, 2 -** **important, 3 - very important** | **Priority for SB (CH): 1 -** **little, 2 -** **important, 3 - very important** | **Priority for ML (DE): 1 -** **little, 2 -** **important, 3 - very important** | **Relevance for which Agri-SSP (SSP1, SSP2 or SSP5): 1 - low, 2-medium, 3- high** | | | **Responsible governance level or institution** | **Evaluation score** |
| --- | --- | --- | --- | --- | --- | --- | --- | --- | --- | --- | --- |
|  |  |  |  |  |  |  | **SSP1** | **SSP2** | **SSP5** |  |  |
| Raising awareness of producers’ & consumers’ social and ecological responsibility for nature & “ecological treasures” (e.g. regarding role of biodiversity, pollinators, farm birds, connectivity of ecological networks at farm and regional level) | 1 | 2 | 3 | 3 | 3 | 2 | 3 | 3 | 1 | educational institutions, nature conservation admin. | 5.75 |
| Raising producers’ & consumers systems knowledge for management and impacts on biodiversity, ecological functions and ecosystem services | 1 | 2 | 3 | 3 | 2 | ND | 3 | 3 | 1 | Ministry of Education, educational institutions | 5.67 |
| Raising consumers' awareness regarding positive aspects of grassland management by adapted livestock heads | 1 | 1 | 2 | 3 | 1 | ND | 3 | 3 | 1 | Ministry of Education, educational institutions | 4.00 |
| Guidelines how to compile landscape management plan at farm level considering biodiversity and ecosystem services (e.g. water protection) | 2 | 2 | 2 | 2 | 1 | 2 | 3 | 2 | 1 | Ministry of Education, educational institutions | 5.75 |
| Training and consultancies regarding IT, technologies and big data and how they can be used to make biodiversity- and environmentally-friendly management decisions | 2 | 2 | 2 | 3 | 1 | 2 | 3 | 3 | 1 | Ministry of Education, educational institutions | 6.00 |
| Certification/labelling of products from heritage or high biodiversity/species-rich meadows or peatlands | 2 | 2 | 3 | 3 | already in place | 2 | 3 | 3 | 1 | state, industry | 6.67 |
| Civil engagement in landscape maintenance | 2 | 2 | 3 | 3 | 3 | 2 | 2 | 3 | 1 | provincial/regional | 6.67 |
| The citizen science approach (e.g. farmers are guided and encouraged to gather nature data from their managed fields, incl. Quality control). | 1 | 2 | 3 | 3 | 3 | 1 | 3 | 2 | 1 | Ministry of Rural Affairs, farmers | 5.50 |
| Biodiversity accounting of products and continuous improvements of the methodology | 2 | 2 | 3 | 2 | already in place | 2 | 3 | 3 | 1 | industry, private organizations, NGOs | 6.33 |
| Championships for land use actions | 2 | 3 | 3 | 3 | 2 | ND | 2 | 3 | 1 | provincial/regional, industry | 7.67 |
| Management standards (e.g. input levels, schedules of fertilizers, pesticides, manure, mowing) | 2 | 3 | 2 | 2 | 3 | 2 | 3 | 2 | 1 | state, industry | 7.67 |
| Law on chemical-synthetical pesticide-free agriculture | 2 | 3 | 2 | 2 | 3 | 2 | 3 | 2 | 1 | state | 7.25 |
| No pesticides for private gardens or public green spaces (e.g. from municipalities) | 2 | 2 | 2 | 2 | 2 | ND | 3 | 3 | 1 | state, provincial/regional | 7.25 |
| Simplification of the leasing system of state land to regional managers | 2 | 3 | Not relevant | 3 | not applicable | 1 | 3 | 3 | 3 | state | 6.00 |
| Spatial planning: designation of grasslands and meadows which may not be taken out of production, e.g. where afforestation, abandonment and other uses (e.g. PV, windparks, soil sealing) should be avoided | 2 | 3 | 3 | 3 | 3 | 2 | 3 | 2 | 1 | provincial/regional | 7.00 |
| Spatial planning: designing and designating ecological networks, valuable cultural landscapes and valuable agricultural land | 2 | 3 | 3 | 3 | 3 | 2 | 3 | 2 | 1 | provincial/regional | 7.75 |
| Establish and finance a long-term biodiversity monitoring | 2 | 3 | 3 | 3 | already in place | 2 | 3 | 2 | 1 | state | 7.75 |
| Input and output taxation (e.g. higher taxes for fertilizers and pesticides, lower VAT rate for biodiversity-friendly agricultural products, higher VAT for products with low biodiversity) | 2 | 2 | 3 | 2 | 3 | 2 | 3 | 2 | 1 | state, EU | 6.50 |
| Private subsidies for biodiversity-friendly production (e.g. from industry or private organizations) | 2 | 2 | 3 | 3 | 2 | 2 | 3 | 2 | 1 | industry, private organizations, NGOs | 6.50 |
| Sale of carbon credits (e.g. carbon markets for farmers) | 1 | 2 | 2 | 2 | 3 | 2 | 3 | 3 | 1 | state, EU | 5.33 |
| Financial compensation for biodiversity monitoring activities of farmers (e.g. via long-term biodiversity monitoring scheme or citizen science approach) | 2 | 2 | 2 | 3 | 2 | 1 | 3 | 3 | 1 | state, provincial/regional | 6.33 |
| Implement result-based agri- environmental programs for preserving and enhancing biodiversity (e.g. based on target/indicator species of pollinators, etc) | 2 | 3 | 3 | 3 | 2 | 2 | 3/0* | 3 | 1 | state | 7.50 |
| Subsidies for higher variations of cultivated crops and livestock, especially for rare and old varieties | 2 | 3 | 2 | 2 | 2 | 2 | 3 | 3 | 1 | state | 7.00 |
| Improved financial support of protected areas and agri-environment-climate measures within agri-environmental programs | 3 | 3 | 3 | 3 | 3 | 3 | 3 | 3 | 1 | state | 9.00 |
| Innovative, well-numerated nature conservation contracts | 2 | 3 | 2 | ND | 2 | 3 | 3 | 3 | 2 | state, provincial/regional | 7.33 |

Additional LBAs for Läänemaa:

Table SM.B 5.2: Case study specific LBAs for Läänemaa for SSP1

| **Type of LBA** | **LBA description** | **Applica-bility: 0 - not possible, 1 - difficult, but possible, 2 - easily applicable** | **Impact on biodiversity: 1 -** **little, 2 -** **medium, 3 - substantial** | **Prioriy for** **Läänemaa: 1 -** **little, 2 -** **important, 3 - very important** | **Responsible governance level or institution** | **Comments** | **Summary evaluation of the LBAs** |
| --- | --- | --- | --- | --- | --- | --- | --- |
| Info | •Econet: Awareness raising on linkages between landscape elements and ecosystem services | 1 | 3 | 2 |  |  | 6 |
|  | •Econet: Guidelines how to compile landscape management plan at farm level considering biodiversity and water protection | 2 | 3 | 2 |  | these topics should already be covered in vocational schools by the agricultural department. in specialties | 7 |
|  | •Paludiculture: championships for farmers successfully managing peatland soils | 2 | 3 | 3 | Estonian Wetlands Association, Ministry of Education, Estonian Fund for |  | 8 |
|  | •Paludiculture: Actions that benefit the farmers' income and environment, e.g. sustainable peatlands management ecolabelling. | 2 | 3 | 3 | Estonian Environment Agency | A product specific to Lääne County. Find new places to grow reeds. | 8 |
|  | •General: Market benefits for Läänemaa - diversification of regional products, organic production, products from heritage meadows | 2 | 3 | 3 |  |  | 8 |
|  | •General: Cooperation between different private sector initiatives, e.g. farms, SPAs, agrotourism |  |  |  | Lääne LEADER, SA Läänemaa tourism |  |  |
| Legal | •Econet: Spatial planning designating ecological networks, valuable cultural landscapes and valuable agricultural land | 1 | 3 |  | Ministry of Financial Affairs, regional governments | Nature conservation admin should be aware and educated on this topic and they should be allowed to give their expert opinions. | 4 |
|  | •Econet: Designation of grasslands where afforestation of natural grasslands and meadows is avoided | 1 | 3 | 3 |  | Already in progress | 7 |
|  | •Econet: Simplification of the leasing system of state land to regional managers - if the current manager has used the land responsibly, it should be possible to renew the contracts without public tender. | 1 | 3 | 3 |  | state property legislation needs changing | 7 |
|  | •Paludiculture: Permanent grasslands that are located in the Natura 2000 and whose soil is 100% peat are considered to be environmentally sensitive permanent grasslands. In the future, not only permanent grassland with 100% peat soil in a Natura 2000 area but all grassland with 100% peat soil could be listed as environmentally sensitive grassland. | 1 | 3 | 3 |  | is dependent on soil map | 7 |
| Economic | •Econet: Support scheme for organic producers in Estonia and farmers who joined the small producers' scheme in 2015 have been exempted from maintaining permanent grassland. In the future, the above-mentioned producers could also be required to maintain permanent grassland, which would equate aid applicants and further increase the area under grassland. | 2 | 3 | 1 |  | there will be a requirement in the new CAP period | 6 |
|  | •Econet: For semi-natural or valuable grasslands, the area coefficient should be applied - higher coefficient for smaller and isolated areas. Direction should be restoration of the adjacent areas. | 1 | 3 | 3 |  | N islets' additional support measure | 7 |
|  | •Econet/general: markets for species-rich meadow hay | 1 | 3 | 3 |  | hay for pets; hay seed fields? | 7 |
|  | •Paludification: Sale of carbon credits (e.g. carbon markets for farmers). | 1 | 3 | 3 |  |  | 7 |
|  | • General: markets for hay. Find use for hay which is not used for fodder. Currently in Lihula heating plant there is not enough hay as hay is being sold outside of Estonia. |  |  |  |  |  |  |
|  | • General: additional use for areas with peaty soils, e.g. bird observations. |  |  |  |  |  | salbes.eu |

Table SM.B 5.3: Case study specific LBAs for Läänemaa for SSP2

| **Type of LBA** | **LBA description** | **Applica-bility: 0 - not possible, 1 - difficult, but possible, 2 - easily applicable** | **Impact on biodiversity: 1 -** **little, 2 -** **medium, 3 - substantial** | **Prioriy for** **Läänemaa: 1 -** **little, 2 -** **important, 3 - very important** | **Responsible governance level or institution** | **Comments** | **Summary evaluation of the LBAs** |
| --- | --- | --- | --- | --- | --- | --- | --- |
| Info | •General: Awareness raising on “a role of biodiversity - pollinators and farm birds”, how to increase connectivity of ecological networks at farm and regional level | 2 | 2 | 3 | educational institutions, nature conservation admin. | it is of utmost priority, because in Läänemaa we still have nature! | 7 |
|  | •General: Awareness raising on how to increase connectivity of ecological networks at farm and regional level | 1 | 3 | 3 | Ministry of Education, educational institutions (applicative agric. education) |  | 7 |
|  | •General: Championships for biodiversity protection; Actions to inform farmers about the progress and make them proud of their achievements (e.g. engaging volunteers in | 2 | 3 | 3 | Ministry of Education, educational institutions, farmers unions | currently only on protected areas | 8 |
|  | •General: The citizen science approach (e.g. farmers are guided and encouraged to gather nature data from their managed fields). | 1 | 2 | 3 | Ministry of Rural Affairs, farmers | first steps done in agriculture-biodiv research projects: A. Helmi projects, Forests for Grassland project | 6 |
| Legal | • Grasslands: Law on prohibiting of cultivation of heritage meadows | ettevalmistamisel | 2 | 3 | Ministry of Education, parliament, Ministy of Rural Affairs, PRIA, nature conserv. admin. |  | 5 |
| Economic | •General: Implement result-based agri- environmental programs for preserving and enhancing biodiversity (e.g. based on target/indicator species of pollinators, etc) | 1 | 3 | 3 | Ministry of Education + Ministry of Rural Affairs | will be done from 2023 as a pilot project | 7 |
|  | •Grasslands: Species growing or living on special grassland as permanent, endangered or infrequent grassland receive additional support from the producer. The support could be motivating, such as the support for beekeeping areas (193 € / ha). | 1 | 3 | 3 | Ministry of Education + Ministry of Rural Affairs | Species can be difficult to identify, this particular measure is a bee measure and is not directly transferable to species. The complexity also depends on whether silo can be made or not. | 7 |

Table SM.B 5.4: Case study specific LBAs for Läänemaa for SSP5

| **Type of LBA** | **LBA description** | **Applic-ability: 0 - not possible, 1 - difficult, but possible, 2 - easily applicable** | **Impact on biodiversity: 1 -** **little, 2 -** **medium, 3 - substantial** | **Prioriy for** **Läänemaa: 1 -** **little, 2 - important, 3 - very important** | **Responsible governance level or institution** | **Comments** | **Summary evaluation of the LBAs** |
| --- | --- | --- | --- | --- | --- | --- | --- |
| Info | •General: Assessment of the effects of drainage and get an adequate picture and then decide separately where to apply mitigation measures. Cooperation between researchers and business. Mutual interest and will. |  |  |  |  | Cooperation with different fields of researchers (e.g. econ., biodiv) is very important, entrepreneurs should definitely take part in it as well. |  |
|  | On mineral soil, we could talk about a bilaterally adjustable system. It would not be practical to use drainage at all on peat soil. | 1 | 2 | 3 | state, farmers' unions | Cooperation with the mineral soil cooperative could be a good opportunity. Certainly a ditch is better than drainage. | **6** |
|  | It would be important to offer effective solutions to the farmer. Tailor-made solutions could be developed for Lääne County! Regional solutions! |  |  |  |  |  |  |
|  | Every farmer should be able to assess what is happening in his field and make decisions based on it, using IT and digital technologies. Technological solutions help to make smart and thoughtful decisions. | 1 | 3 | 1 | state |  | **5** |
|  | Raising awareness, knowledge of the land must increase. Big data could help. Inclusive education. |  |  |  |  |  |  |
|  | The question of support for heritage meadows |  |  |  |  | Performance-based grant for heritage meadows |  |
| Economic | •General: financial subsidies for creation of solar park as an infrastructure pollinator support area (to be combined with a spatial planning and awareness-raising activity) | 2 | 2 | 2 | state | It would work if the property is owned by a food producing farmer and the land is leased to someone who only uses it for economic purposes and does not care about biodiversity | **6** |
|  | Sale of carbon units | 1 | 2 | 2 | state, responsible are Ministry of Education and MoRural Affairs |  | **5** |
|  | Cultivation of wetlands is important for the region. In some regions, it is definitely necessary to irrigate |  |  |  |  |  |  |
|  | Valuing regional biomass is a topic |  |  |  |  |  |  |
|  | Innovation is needed more- (the cow is put on a solar binding blanket and then she ties it herself) no separate panels are needed! |  |  |  |  |  |  |

The following table shows case study specific LBAs for Münsterland in Germany for all three SSPs.

Table SM.B 5.5: Overview of the LBAs for Münsterland in Germany

| Land-use-biodiversity activity (LBA) | applicability | Impact on Biodiversity & Environment | Priority for Münsterland | Relevance for SSP1 | Relevance for SSP2 | Relevance for SSP5 | Government Level |
| --- | --- | --- | --- | --- | --- | --- | --- |
|  | · 1 not possible | · 1 low | · 1 low | · 1 low | · 1 low | · 1 low | N Federal level |
|  | · 2 difficult | · 2 medium | · 2 medium | · 2 medium | · 2 medium | · 2 medium | BL State Level |
|  | · 3 light applicable | · 3 high | · 3 very important | · 3 high | · 3 high | · 3 high | R Regional level ( administrative district ) |
|  |  |  |  |  |  |  | L Regional level |
| Information and institutions |  |  |  |  |  |  |  |
| 1. Raising awareness among producers and consumers about their social and environmental responsibility for nature and "ecological treasures" | 2 | 2 | 2 | 3 | 3 | 1 | BL, R, L |
| 2. Guidelines for the preparation of farm development plans taking into account biodiversity and ecosystem services (e.g. water protection) | 2 | 2 | 2 | 3 | 2 | 1 | BL, R |
| 3. Training and consulting regarding technologies (agricultural technology, IT, Big Data) and their use for decisions in the field of environmentally friendly management. | 2 | 2 | 2 | 2 | 3 | 3 | BL |
| 4. Certification of regional products from areas with high species richness or great biodiversity | 2 | 2 | 2 | 3 | 2 | 1 | BL, R |
| 5. Civic engagement in landscape conservation | 2 | 2 | 2 | 3 | 2 | 1 | R, L |
| 6. Citizen Science approach (e.g., farmers collect nature data from their cultivated fields) | 2 | 1 | 1 | 3 | 2 | 1 | N, BL |
| 7. Accounting for the biodiversity of products and continuous improvement of the methodology | 2 | 2 | 2 | 3 | 2 | 1 | N, BL |
| Legal norms |  |  |  |  |  |  |  |
| 1. Management standards (e.g., level of fertilizer, pesticide, manure, mowing) | 2 | 3 | 2 | 3 | 2 | 2 | N, BL |
| 2. Law on chemical-synthetic pesticide-free agriculture | 2 | 2 | 2 | 3 | 1 | 1 | N, BL |
| 3. Simplification of the system for leasing state-owned land to regional managers (with responsible use by the current tenant, renewal of contracts without public tender) | 2 | 2 | 1 | 3 | 1 | 1 | N, BL |
| 4. Spatial planning: Designation of grassland and meadows that must not be taken out of production, e.g., where afforestation, set-aside and other uses (e.g., PV, wind farms, soil sealing) should be avoided. | 2 | 2 | 2 | 3 | 2 | 2 | BL, R, L |
| 5. Spatial planning: Design and designation of ecological networks, valuable cultural landscapes and valuable agricultural areas | 2 | 2 | 2 | 3 | 2 | 1 | BL, R, L |
| 6. Establishment and funding of long-term biodiversity monitoring: Expand monitoring | 2 | 2 | 2 | 3 | 2 | 1 | N, BL |
| Economic instruments |  |  |  |  |  |  |  |
| 1. Input and output taxation (taxation based on contribution to biodiversity promotion) | 2 | 2 | 2 | 3 | 2 | 2 | N |
| 2. Private subsidies for biodiversity-friendly production (e.g. from industry or private organizations) | 2 | 2 | 2 | 3 | 2 | 2 | N, BL, R, L |
| 3. Sale of carbon credits (e.g., carbon markets for farmers) | 2 | 2 | 2 | 3 | 3 | 2 | N |
| 4. Financial compensation for farmers monitoring biodiversity (e.g., as part of a long-term monitoring program) | 2 | 2 | 1 | 3 | 3 | 2 | N, BL |
| 5. Implementation of results-oriented agri-environmental programs to conserve and promote biodiversity (e.g. based on target/indicator species of pollinators, etc.) | 2 | 3 | 2 | 3 | 2 | 2 | N, BL |
| 6. Subsidies for greater diversity of cultivated plants and farm animals (rare and old varieties) | 2 | 2 | 2 | 3 | 3 | 2 | N, BL |
| 7. Increased financial support for protected areas and agri-environmental and climate measures within the framework of agri-environmental programs. | 3 | 3 | 3 | 3 | 3 | 2 | N, BL |
| 8. innovative, well -paid nature conservation agreements | 2 | 3 | 3 | 3 | 3 | 1 | N, BL |

## References

Agricultural Registers and Information Board, PRIA, 2020

Conradt, T., 2021. SALBES Deliverable Report Deliverable 3.1 - Climate scenario report for the case study regions (No. 3.1 v2.0).

Durán, A.P., Kuiper, J.J., Aguiar, A.P.D., Cheung, W.W.L., Diaw, M.C., Halouani, G., Hashimoto, S., Gasalla, M.A., Peterson, G.D., Schoolenberg, M.A., Abbasov, R., Acosta, L.A., Armenteras, D., Davila, F., Denboba, M.A., Harrison, P.A., Harhash, K.A., Karlsson-Vinkhuyzen, S., Kim, H., Lundquist, C.J., Miller, B.W., Okayasu, S., Pichs-Madruga, R., Sathyapalan, J., Saysel, A.K., Yu, D., Pereira, L.M., 2023. Bringing the Nature Futures Framework to life: creating a set of illustrative narratives of nature futures. Sustain Sci. https://doi.org/10.1007/s11625-023-01316-1

Kim, H., Peterson, G.D., Cheung, W.W.L., Ferrier, S., Alkemade, R., Arneth, A., Kuiper, J.J., Okayasu, S., Pereira, L., Acosta, L.A., Chaplin-Kramer, R., den Belder, E., Eddy, T.D., Johnson, J.A., Karlsson-Vinkhuyzen, S., Kok, M.T.J., Leadley, P., Leclère, D., Lundquist, C.J., Rondinini, C., Scholes, R.J., Schoolenberg, M.A., Shin, Y.-J., Stehfest, E., Stephenson, F., Visconti, P., van Vuuren, D., Wabnitz, C.C.C., José Alava, J., Cuadros-Casanova, I., Davies, K.K., Gasalla, M.A., Halouani, G., Harfoot, M., Hashimoto, S., Hickler, T., Hirsch, T., Kolomytsev, G., Miller, B.W., Ohashi, H., Gabriela Palomo, M., Popp, A., Paco Remme, R., Saito, O., Rashid Sumalia, U., Willcock, S., Pereira, H.M., 2023. Towards a better future for biodiversity and people: Modelling Nature Futures. Global Environmental Change 82, 102681. https://doi.org/10.1016/j.gloenvcha.2023.102681

Suškevičs, M., Karner, K., Bethwell, C., Danzinger, F., Kay, S., Nishizawa, T., Schuler, J., Sepp, K., Värnik, R., Glemnitz, M., Semm, M., Umstätter, C., Conradt, T., Herzog, F., Klein, N., Wrbka, T., Zander, P., Schönhart, M., 2023. Stakeholder perceptions of agricultural landscape services, biodiversity, and drivers of change in four European case studies. Ecosystem Services 64, 101563. https://doi.org/10.1016/j.ecoser.2023.101563

Villoslada, M., Vinogradovs, I., Ruskule, A., Veidemane, K., Nikodemus, O., Kasparinskis, R., Sepp, K., Gulbinas, J., 2018. A multitiered approach for grassland ecosystem services mapping and assessment: The Viva Grass tool. One Ecosystem 3, e25380. https://doi.org/10.3897/oneeco.3.e25380
